# QTL mapping and transcriptome analysis identify novel QTLs and candidate genes in *Brassica villosa* for quantitative resistance against *Sclerotinia sclerotiorum*

**DOI:** 10.1007/s00122-023-04335-9

**Published:** 2023-03-26

**Authors:** Thomas Bergmann, Jan Menkhaus, Wanzhi Ye, Markus Schemmel, Mario Hasler, Steffen Rietz, Gunhild Leckband, Daguang Cai

**Affiliations:** 1 Department of Molecular Phytopathology and Biotechnology, Institute of Phytopathology, Christian-Albrechts-University of Kiel, Hermann-Rodewald-Str. 9, 24118 Kiel, Germany; 2 Lehrfach Variationsstatistik, Christian-Albrechts-University of Kiel, 24118 Kiel, Germany; 3 NPZ Innovation GmbH, 24363 Holtsee, Germany; 4 Present Address: NPZ Innovation GmbH, 24363 Holtsee, Germany

## Abstract

**Key message:**

**Novel QTLs and candidate genes for Sclerotinia-resistance were identified in** ***B. villosa*** **, a wild Brassica species, which represents a new genetic source for improving oilseed rape resistance to SSR.**

**Abstract:**

Sclerotinia stem rot (SSR), caused by *Sclerotinia sclerotiorum,* is one of the most destructive diseases in oilseed rape growing regions. To date, there is no effective genetic resistance against *S. sclerotiorum* in the *B. napus* germplasm and knowledge of the molecular plant–fungal interaction is also limited. To identify new resistance resources, we screened a set of wild Brassica species and identified *B. villosa* (BRA1896) with a high level of Sclerotinia-resistance. Two segregating *F*_2_ populations for Sclerotinia-resistance, generated by interspecific crosses between the resistant *B. villosa* (BRA1896) and the wild susceptible *B. oleracea* (BRA1909) were assessed for Sclerotinia-resistance. Genetic mapping using a 15-k Illumina Infinium SNP-array resulted in a high-density genetic map containing 1,118 SNP markers and spanning a total genetic length of 792.2 cM. QTL analysis revealed seven QTLs explaining 3.8% to 16.5% of phenotypic variance. Intriguingly, RNAseq-based transcriptome analysis identified genes and pathways specific to *B. villosa,* of which a cluster of five genes encoding putative receptor-like kinases (RLKs) and two pathogenesis-related (PR) proteins are co-localized within a QTL on chromosome C07. Furthermore, transcriptomic analysis revealed enhanced ethylene (ET)-activated signaling in the resistant *B. villosa,* which is associated with a stronger plant immune response, depressed cell death, and enhanced phytoalexin biosynthesis compared to the susceptible *B. oleracea.* Our data demonstrates that *B. villosa* represents a novel and unique genetic source for improving oilseed rape resistance against SSR.

**Supplementary Information:**

The online version contains supplementary material available at 10.1007/s00122-023-04335-9.

## Introduction

The necrotrophic fungus *Sclerotinia sclerotiorum* (Lib.) de Bary, a soil-borne fungal pathogen, causes the disease Sclerotinia stem rot (SSR) in rapeseed oil. The fungus embraces a broad spectrum of host plants with more than 400 species including many economically important crops (Boland and Hall 1994; Bolton et al. 2006). *S. sclerotiorum* overwinters as sclerotia in the soil which are the main inoculum for SSR epidemics in oilseed rape. In spring, apothecia growing on sclerotia carpogenically germinate producing ascospores that are released into air currents and deposited to aboveground parts of the host plant such as the stem axils. When conditions are favorable the fungus starts to grow and infect healthy stem tissue (Derbyshire and Denton-Giles 2016). The pronounced virulence of the fungus is among others attributed to a broad repertoire to produce cell wall-degrading enzymes, phytotoxins, and secreted effector proteins (Amselem et al. 2011). Most common symptoms are bleached lesions traversed by white mycelium in the stem or branch and the formation of black sclerotia inside the infected tissue (Bolton et al. 2006). SSR is one of the most destructive diseases in many oilseed rape cultivation areas worldwide. The tremendous increase in cultivation area of oilseed rape in combination with shorter crop rotation cycles favored SSR dispersal over the past decades. Stems of infected plants tend to burst and shatter. The weakened stem stability and resulting lodging of the plants can cause severe yield and quality losses in oilseed rape cultivation (Derbyshire and Denton-Giles 2016). Resistance to Sclerotinia is mainly measured via leaf-, petiole-, or stem-inoculations on the basis of Zhao et al. (2004) and Zhao and Meng (2003). Studies attempting to assess correlations between the different resistance traits reported contradictory results (Mei et al. 2011, 2013; Taylor et al. 2018; Uloth et al. 2013; You et al. 2016) and more effort is needed to determine their genetic link. Though SSR can be effectively controlled by application of fungicides, the increasing restriction of fungicide use due to its potential environmental and health hazards and the emergence of resistant isolates ask for alternative control strategies worldwide (Derbyshire and Denton-Giles 2016; Wang et al. 2014; Zhou et al. 2014).

Breeding for resistant varieties is an important method in plant disease management. So far, no effective genetic resistance against SSR is available in the *B. napus* gene pool (Derbyshire and Denton-Giles 2016). Few *B. napus* genotypes that feature partial Sclerotinia-resistance were reported (Li et al. 2009b; Wang et al. 2002; Zhang et al. 2002). QTLs for Sclerotinia-resistance were also identified in various *B. napus* mapping populations (Behla et al. 2017; Wei et al. 2014; Wu et al. 2013; Yin et al. 2010; Zhao et al. 2006; Zhao and Meng 2003) and genome-wide association studies (GWAS) identified single-nucleotide polymorphisms (SNPs) associated with Sclerotinia-resistance in numerous *B. napus* accessions (Gyawali et al. 2016; Wei et al. 2016; Wu et al. 2016).

Therefore, efforts have been made to transfer high SSR-resistance from interspecific crosses to the primary gene pool of *B. napus* (Chen et al. 2007; Garg et al. 2010). The *Brassica oleracea* complex, including *B. incana*, *B. rupestris*, *B. insularis* and *B. villosa*, was identified as valuable pool of high Sclerotinia-resistance (Mei et al. 2011; Taylor et al. 2018). Mei et al. (2013) identified QTLs for SSR-resistance in a mapping population from an interspecific cross between the wild *B. incana* (resistant) and the cultivated *B. oleracea* var. *alboglabra* (susceptible). The resistance was partially transferred into the *B. napus* gene pool (Mei et al. 2015, 2020). The transcriptome analysis of *B. incana* linked the resistance to an increased oxidative burst at the infection site balanced by Ca^2+^ signaling and a suppressed degradation of the plant cell wall by *S. sclerotiorum* (Ding et al. 2019). These studies highlight the *B. oleracea* gene pool as important source for introgression of improved resistance to Sclerotinia into the primary gene pool of *B. napus*.

In this study, we report genetic mapping and QTL analysis of Sclerotinia-resistance in the wild cabbage species *Brassica villosa* and present for the first time the QTLs for Sclerotinia-resistance in this genome. The comparison with previously identified QTLs in the wild *B. incana* (Mei et al. 2013) allows for evaluating the resistance mechanisms existing in different Brassica species. In addition, transcriptome analysis was performed to provide molecular insights into the early defense response in *B. villosa*. We identified 58 defense-related genes to be specifically upregulated in the resistant *B. villosa* in response to Sclerotinia-inoculation and a cluster of five putative RLKs and two PR proteins which are co-localized within one QTL on the chromosome C07 of the *B. oleracea* reference genome. Furthermore, the transcriptome analysis suggests that the distinct activation of signaling pathways mediated by ET may play a pivotal role in the Sclerotinia-resistance that is associated with a strong immune response, a negative regulation of cell death, and an elevated phytoalexin biosynthesis in *B. villosa*.

## Materials and methods

### Plant material and population structure

Seeds of wild Brassica species (BRA1909, BRA2886, BRA3044, BRA2856, BRA2945, BRA1166, BRA2923, BRA1262, BRA2918, BRA1896) were obtained from the Institute of Plant Genetics and Crop Plant Research (Gatersleben, Germany) and screened for Sclerotinia-resistance with the detached leaf- and petiole-assays (Mei et al. 2011; Zhao et al. 2004) under greenhouse conditions. From this set, the highly resistant wild species *B. villosa* (BRA1896) was crossed with the susceptible *B. oleracea* (BRA1909) (Supplementary Data S1). Two mapping populations with 252 and 258 *F*_2_ individuals (referred to as Population A and B) were cultivated under greenhouse conditions in two separate years, of which 234 and 258 *F*_2_ individuals were screened for Sclerotinia-resistance. For each population, two 15-k Brassica SNP-chip assays (TraitGenetics, Gatersleben, Germany) were used with each chip carrying a total of 96 samples including two NTCs. Therefore, we selected 187 and 184 *F*_2_ individuals for genotyping from Population A and B, respectively. The remaining slots on the chips were reserved for genotyping of the parental species. The 184 *F*_2_ genotyped individuals from Population B were re-evaluated twice for Sclerotinia-resistance.

### Resistance screening and population comparison

Resistance evaluation was performed with the leaf- and petiole-inoculation (Mei et al. 2011; Zhao et al. 2004). Though the stem-inoculation is the most comparable assay to the natural Sclerotinia infection, early and late flowering phenotypes which started to shoot when other plants were already in senescence as well as diverse morphology made an evaluation for stem-resistance difficult and was therefore not performed in our mapping populations. We used a *S. sclerotiorum* strain originally isolated from an oilseed rape field in Chongqing, China (Mei et al. 2011). The fungus was cultured on potato dextrose agar (PDA; 20 g/l PDB, 15 g/l Bacto agar) plates with a pH of 5.6 at 21 °C and transferred to a new PDA plate two days before inoculation with a cork corer (Ø 8 mm). Leaf resistance was evaluated with at least three Sclerotinia-plugs on detached leaves with the mycelia-site facing the leaf surface. The third and fourth leaves (counted from the apical meristem) and their detached petioles were used for inoculation. Petiole resistance was evaluated with Sclerotinia-plugs stuck on the open cut of the petioles with 1-ml pipette tips. Detached leaves and petioles were placed in a tray with wetted paper towel placed around open cut surfaces and sealed with foil. Leaf-lesion area and petiole-lesion length were measured at 2 days post-infection (dpi). The leaf-lesion area was calculated with the following equation:

$${\text{Leaf-lesion}}\;{\text{area}}\left[ {{\text{mm}}^{2} } \right] = \pi \cdot a \cdot b$$

‘a’ equals the semi-major axis and ‘b’ indicates the semi-minor axis of a lesion ellipse. The mean leaf- and petiole-lesion value was calculated for each individual plant. Lesion values of the parental plants were used to characterize *F*_2_ individuals for their resistance in the whole population screenings. Plants with lesion values smaller than that of the resistant parent (*B. villosa*) were classified as resistant while plants with lesion values larger than that of the susceptible parent (*B. oleracea*) were classified as susceptible. Plants with lesion values between those of *B. villosa* and *B. oleracea* were defined as intermediate. All statistical analyses were performed via the R software (R Core Team 2021). Data handling in R was mainly performed with the dplyr package (Wickham et al. 2021). Parental lesions in each population were compared via a linear model. Analysis of variance (ANOVA) was followed by multiple contrast tests with the multcomp package (Hothorn et al. 2008). Pearson’s correlation analysis was performed between leaf- and petiole-lesions in each inoculation-assay. Figures were created via the ggplot2 package (Wickham 2016). The phenotype data is available in the Supplementary Data S2–S4.

### Trypan blue staining

Detached leaves of *B. villosa* and *B. oleracea* were placed in petri dishes, inoculated with PDA-plugs of actively growing Sclerotinia and sealed with Parafilm. After 2 dpi, leave tissue of the junction between necrotic and non-necrotic material was hand-dissected in small rectangles, placed into petri dishes and stained with Trypan blue staining solution according to Fernández-Bautista et al. (2016). Samples were visualized and taken with a SteREO Discovery.V20 microscope (Carl Zeiss AG, Oberkochen, Germany), an AxioCam MRc microscope-cam (Carl Zeiss AG, Oberkochen, Germany), and the AxioVision software (Carl Zeiss AG, Oberkochen, Germany).

### Genotyping and genetic map construction

DNA was isolated from leaves following the cetyltrimethylammonium bromide (CTAB) method (Rogers and Bendich 1985). DNA concentration was adjusted to 20 ng/µl with 1% agarose gel with Lambda-DNA (Thermo Fisher Scientific, Massachusetts, USA) and the Gel Doc™ Image Lab Software (Bio-Rad Laboratories, California, USA). Plants were genotyped by TraitGenetics (Gatersleben, Germany) with the 15-k Brassica SNP-chip assay (TraitGenetics, unpublished). The chip carries a total of 13,714 SNP markers. We evaluated the publicly available Brassica reference genomes by RNAseq read-based alignment analysis and found that the genome of *B. oleracea* (cv. TO1000) represents the best reference genome for this study. The SNP marker sequences were searched against the *B. oleracea* TO1000 reference genome (Parkin et al. 2014) via the BLAST + software (Altschul et al. 1990; Camacho et al. 2009) with the following options: -evalue 1e−5; -max_target_seqs 2; -max_hsps 1; -outfmt 6. SNPs were transformed into ABH alleles via custom-written python code. Genotypes of both mapping populations were merged for construction of a common genetic map to enable a better comparison of QTLs between both mapping populations which had no influence on the QTL analysis itself. The genetic map was constructed with the R/qtl package (Broman et al. 2003). The linkage groups were assigned to the chromosomes of the *B. oleracea* TO1000 reference genome via the best hits from the BLAST + search. The genetic and physical positions of all SNP marker are available in Supplementary Data S5.

### QTL mapping

The QTL analysis was performed with the R/qtl package according to the workflow described in Broman and Sen (2009) separately for each mapping population. A single-QTL model scan (‘scanone’ function) was performed with the Haley–Knott regression (Haley and Knott 1992) followed by a scan with a nonparametric model which considers the rank-based phenotypes (model = ‘np’) when the first scan detected no QTLs. Peak markers of identified QTLs were used as covariates in the single-QTL model to scan for additive and interactive effects of these markers to other loci followed by a two-dimensional QTL scan considering epistatic effects. A multiple-QTL model was set up according to the identified loci from the scans and screened for additional (‘addqtl’ function) and interacting (‘addint’ function) QTLs. The model was adjusted and finally fitted with the forward/backward model selection algorithm with the Haley–Knott method via the ‘stepwiseqtl’ function. The effect and amount of explainable phenotypical variance by each QTL was estimated with an ANOVA of the final multiple-QTL model. QTL intervals were estimated with the 95% Bayes credible interval method in R/qtl. QTLs with overlapping intervals were classified as common QTL. The significance thresholds were determined via genome-scan-adjusted *P* values based on permutation tests (10,000 permutations for the single-QTL scans; 2000 permutations for the two-dimensional scans). The mapping data is available in Supplementary Data S6.

### Library preparation and RNA sequencing

RNA was isolated with the innuPREP Plant RNA Kit (Analytik Jena AG, Jena, Germany) of Sclerotinia-inoculated and mock-treated petioles from *B. villosa* and *B. oleracea* according to the manufacturer’s recommendation. For this, three independent biological replications of each sample were harvested at 8 h post-inoculation (hpi) and immediately frozen in liquid nitrogen. Samples were kept at − 80 °C until further processing. One biological replication consisted of pooled material of eight petioles (1 cm in length from the inoculation site) from four plants. For the transcriptome analysis, we chose 8 hpi to study the early transcriptome response. As we observed that at this time point, the petioles of the resistant plants showed no clear symptoms, while the petioles of the susceptible plants already exhibited profound necrotic lesions at the inoculation sites. This is in accordance with Rietz et al. (2012) and Ding et al. (2019). RNA quality and concentration were determined on 1.3% agarose gel as well as with the NanoVue Plus Spectrophotometer (GE Healthcare, Illinois, USA). Samples were sent to Novogene (Beijing, China) for library preparation and sequencing on the Illumina HiSeq 4000 system. Adapters and reads containing unknown nucleotides (> 10%) and low-quality bases (*Q*-score ≤ 5) with more than 50% of the total bases were removed by Novogene. Raw sequencing data is available at the NCBI Sequence Read Archive (PRJNA706136).

### RNAseq analysis

Data analysis was performed with reference-based and de novo-based RNAseq software tools to bypass limitations of reference-based transcriptome analysis and to provide a more detailed insight into the transcriptome profiles of the wild Brassica species. Briefly, raw reads were processed by removing reads with an average quality less than Q30 (AVGQUAL: 30) via the Trimmomatic software (Bolger et al. 2014). Clean reads were aligned to the *B. oleracea* TO1000 reference genome and assembled to a reference transcriptome via the ‘new Tuxedo’ protocol including HISAT2 (Kim et al. 2019) and StringTie (Pertea et al. 2015) according to Pertea et al. (2016). SAM files were sorted and converted to BAM files via SAMtools (Li et al. 2009a). The GFF utilities gffread and gffcompare (Pertea and Pertea 2020) were used to extract reference transcript sequences and to retrieve transcriptome assembly statistics. The reference gene count matrix was extracted with the enclosed python script in the StringTie software package. Unmapped reads from the reference transcriptome assembly were then extracted from the BAM files via SAMtools (‘samtools view’ command) with the following parameters: − *f* 12; − *F* 256. Unmapped BAM files were converted to fastq format via the ‘bamtofastq’ utility from BEDtools (Quinlan and Hall 2010) and re-aligned to the *S. sclerotiorum* 1980 reference genome (Amselem et al. 2011). Unmapped reads that neither aligned to *B. oleracea* nor to *S. sclerotiorum* were then re-converted to fastq format and assembled de novo via Trinity (Grabherr et al. 2011). Counts of de novo transcripts were estimated via RSEM (Li and Dewey 2011) and the count matrix was filtered for transcripts with at least 10 counts in each of the three biological replications of each sample. Left-over transcripts were loosely defined as genes and merged with the gene count matrix from the reference transcriptome assembly. Differentially expressed gene (DEG) analysis was performed with the merged raw count matrix via the DESeq2 software (Love et al. 2014). The model included the comparison of Sclerotinia-inoculated samples to mock-treated samples of each genotype as well as the interaction term. Genes were considered to be statistically differentially expressed with an adjusted *P* value ≤ 0.05. Regularized (rlog)-transformed samples were checked with the sample-to-sample distance matrix and the principal component analysis (PCA) in DESeq2. Graphical illustrations and data handling were mainly performed in R via the ggplot2 and the dplyr packages. The RNAseq gene table is available in Supplementary Data S7.

### Gene annotation and Gene Ontology (GO) enrichment analysis

The major isoform, measured by the highest averaged FPKM value of all isoforms for a gene across all samples, was used for in silico gene annotation and GO enrichment analysis. The TransDecoder software (https://github.com/TransDecoder/TransDecoder/wiki) was used to convert transcript sequences into protein sequences and to identify functional protein domains. The ‘TransDecoder.LongOrfs’ tool was used to predict longest open reading frames (ORFs) which were then used for a homology-based coding region identification in Pfam (El-Gebali et al. 2019) via the HMMER software (http://hmmer.org/) and in a protein sequence database of Arabidopsis (organism: 3702) downloaded from Uniprot (The UniProt Consortium 2019) via BLASTp + and the following options: -evalue 1e5; -max_target_seqs 1; -max_hsps 1; -outfmt 6. The Pfam and BLAST + results were integrated into the final coding prediction with the ‘TransDecoder.Predict’ tool. The closest homologs in *A. thaliana* were used for GO annotations of the Brassica genes via the KOBAS database from Xie et al. (2011). Additionally, all genes were searched via BLAST + against the *B. napus* Refseq database (taxid: 3708) downloaded from the National Center for Biotechnology Information (NCBI; https://www.ncbi.nlm.nih.gov/). GO enrichment analysis was performed with the goseq package (Young et al. 2010) taking the gene length bias of RNAseq into account. The *P* values were adjusted via the FDR method by Benjamini and Hochberg (1995) and GO terms were considered to be significant with a FDR ≤ 0.05. The comparative GO analysis was performed and output tables were created with custom-written *R*-scripts. The GO enrichment results for biological processes is available in Supplementary Data S8.

### Real-time quantitative PCR (RT-qPCR) analysis

For RT-qPCR, RNA was isolated at 8 hpi and 16 hpi as described before from three independent biological replications of Sclerotinia- and mock-treated petioles from *B. villosa* and *B. oleracea*. Experimental design was identical to the RNAseq experiment. Synthesis of cDNA was performed with 500 µg of RNA treated with RNAse-free DNase I (Thermo Fisher Scientific, Massachusetts, USA) in a volume of 20 µl with the RevertAid First Strand cDNA Synthesis Kit (Thermo Fisher Scientific, Massachusetts, USA) according to the manufacturer’s instructions. Primer targets were checked by PRIMER-BLAST (Ye et al. 2012). Two microliters (1:5 diluted) were mixed with 18 µl Master Mix as described in the manual of the qPCRBIO SyGreen Mix (PCR Biosystems Inc., Pennsylvania, USA). RT-qPCR was performed on a CFX96 Touch Real-Time PCR Detection System (Bio-Rad Laboratories, California, USA). Conditions for the reactions were as follows: 3 min at 95 °C; 40 cycles of 95 °C for 15 s, 60 °C for 15 s, and 72 °C for 20 s. Gene expression was normalized to *BolACT7* (Bo3g005290) and relative quantification between Sclerotinia- and mock-treated samples was calculated according to Pfaffl (2001). The stable expression of *BolACT7* was checked by RNAseq. Analysis of primer efficacy was determined by a standard curve of pooled cDNA from all samples for each gene. Information about primer is provided in the Supplementary Table S1. Statistical analysis was performed via a linear model using generalized least squares with the nlme package (Pinheiro et al. 2021) which included the factors genotype, treatment and hpi, as well as their interaction terms. The residuals were assumed to be normally distributed and to be heteroscedastic based on a graphical residual analysis. ANOVA was conducted and followed by multiple contrast tests with the multcomp package (Hothorn et al. 2008). Sanger sequencing of selected templates at Eurofins Scientific SE (Luxembourg, Luxembourg) in Hamburg, Germany, validated targets in the wild Brassica species.

## Results

### Identification of *B. villosa* as source of high Sclerotinia-resistance

**Fig. 1:**
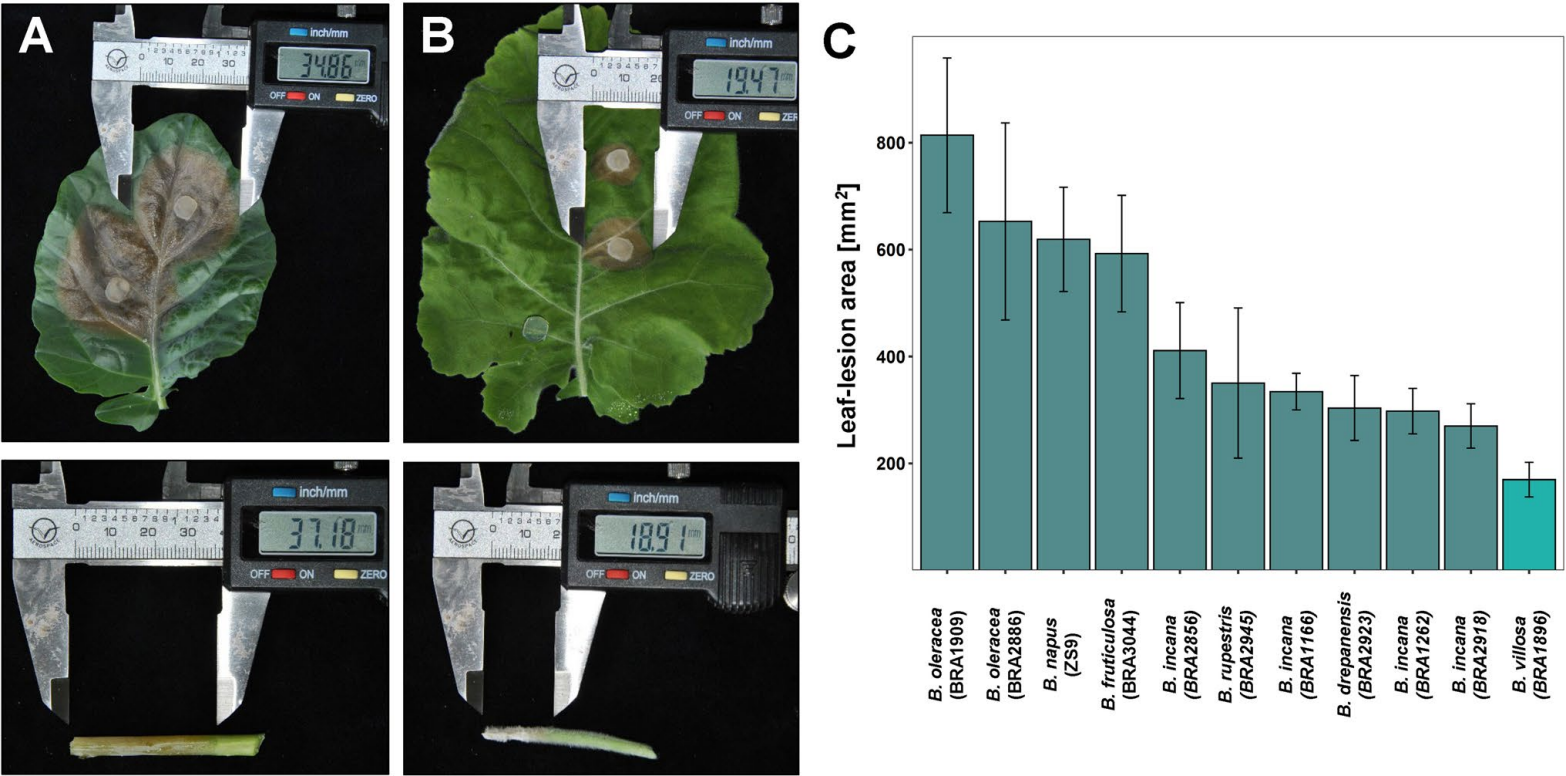
Detached leaf- and petiole-assay of wild Brassica species. **A** Leaf- (top) and petiole- (bottom) lesions of the susceptible *B. oleracea* (BRA1909) and **B** leaf- (top) and petiole- (bottom) lesions of the resistant *B. villosa* (BRA1896). Lesion sizes were measured 2 days post-inoculation (dpi). **C** Leaf-lesion sizes of a collection of wild Brassica species from the gene bank of the Institute of Plant Genetics and Crop Plant Research in Gatersleben, Germany. Lesion sizes were measured at 2 dpi. Error bars represent standard errors from three biological replicates

To evaluate the potential of wild Brassica species for Sclerotinia-resistance, we screened a set of wild Brassica species for their resistance with the widely established leaf- and petiole-inoculation assays (Mei et al. 2011; Zhao et al. 2004) under greenhouse conditions. Because the wild Brassica species used in this study exhibited a high degree of diversity in stem development, as described by Taylor et al. (2018), the stem-inoculation method was not feasible for this study. As a result, *B. villosa* (BRA1896) was identified to be highly resistant to Sclerotinia infection as compared with *B. oleracea* (BRA1909; Fig. 1A, B) and four *B. incana* species (BRA1166, BRA1262, BRA2856, BRA2918; Fig. 1C). In support for this, we observed noticeable differences in the fungal spread on infected leaves between the resistant BRA1896 and the susceptible BRA1909 plants by Trypan blue staining assays. A dense and compact structured growth mainly within the necrotic tissue with a sharply delimited junction between healthy and infected tissue was characteristic for the susceptible *B. oleracea* (Supplementary Figure S1). In the resistant *B. villosa*, the fungal expansion was less structured, mainly centered on the leaf surface with no sharply delimited changeover between healthy and infected tissue and strongly pronounced infection cushions. For genetic analysis of Sclerotinia-resistance in *B. villosa* (BRA1896), two segregating *F*_2_ populations (Population A, Population B) were generated from an interspecific cross between the resistant *B. villosa* (BRA1896) and a wild susceptible *B. oleracea* (BRA1909) in two years.

### Evaluation of ***F***_2_ populations for Sclerotinia-resistance

**Fig. 2:**
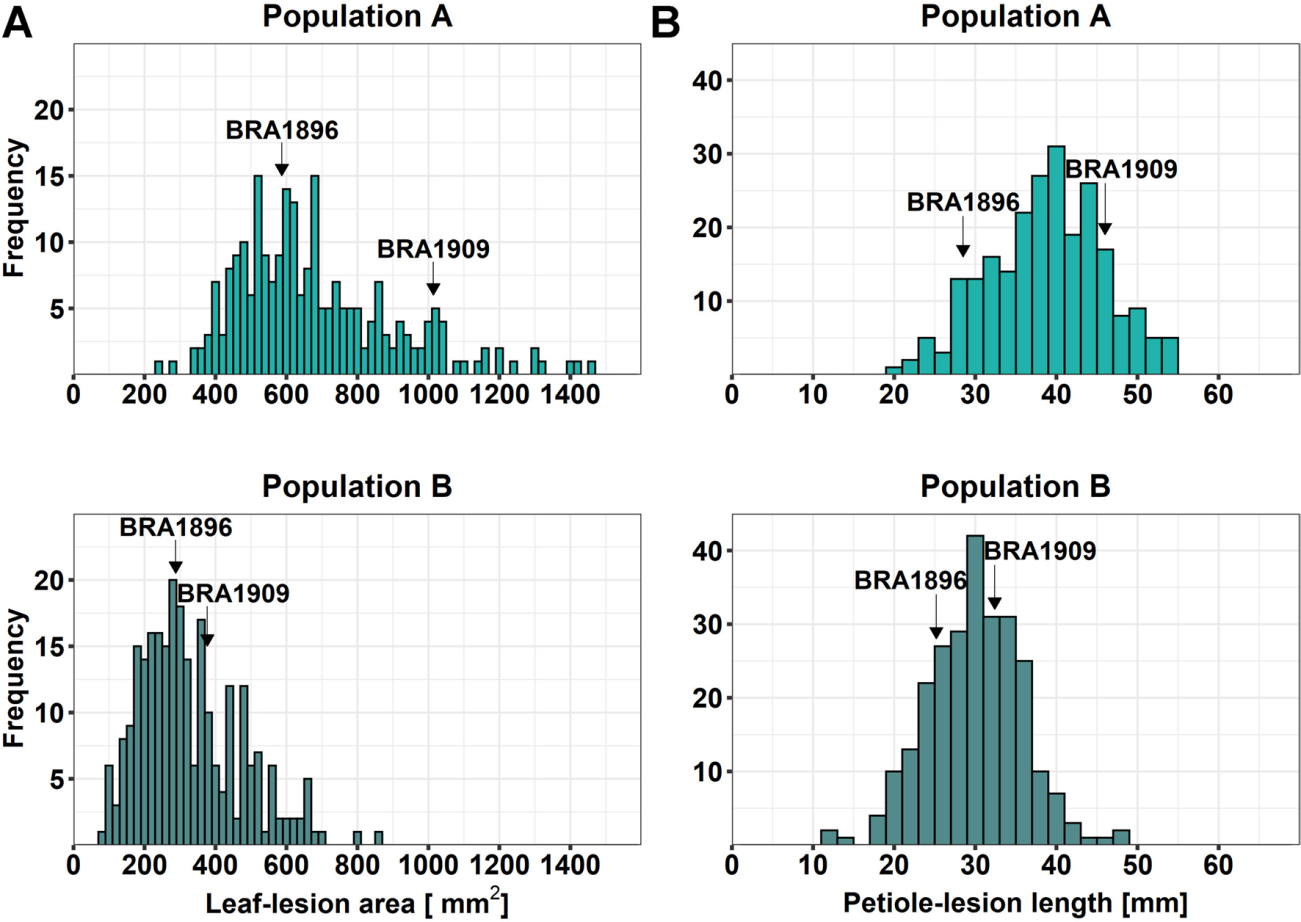
Lesion size distributions in two *F*_2_ mapping populations from a cross between *B. villosa* (BRA1896) and *B. oleracea* (BRA1909). **A** Leaf-lesion size and **B** petiole-lesion size distribution in Population A (top, light green) and Population B (bottom, dark green). Positions of *B. villosa* (BRA1896) and *B. oleracea* (BRA1909) are indicated by arrows

In total, 234 *F*_2_ plants in Population A and 258 *F*_2_ plants in Population B were separately evaluated for Sclerotinia-resistance with the detached leaf- and petiole-assay, respectively. Leaf-lesion values ranged from 241 to 1452 mm^2^ in Population A and from 78 to 867 mm^2^ in Population B, reflecting a slower disease development in the leaf assay in the second year (Fig. 2A.) The petiole-assays produced similar lesion size distributions in the two populations, ranging from 19 to 55 mm in Population A and from 13 to 48 mm in Population B (Fig. 2B). In each population, we compared the leaf- and petiole-lesion sizes of the *F*_2_ plants with the parental plants and divided individual *F*_2_ plants into three categories (resistant, intermediate, susceptible) and compared the numbers of plants showing a similar response in both assays. Thereby, we identified 15 *F*_2_ individuals from Population A and 32 *F*_2_ individuals from Population B, which showed a higher resistance level than *B. villosa* in both assays (Supplementary Figure S2). In total, we identified 207 *F*_2_ plants from both populations which were classified into the same category of resistant, intermediate, and susceptible in both assays.

**Fig. 3:**
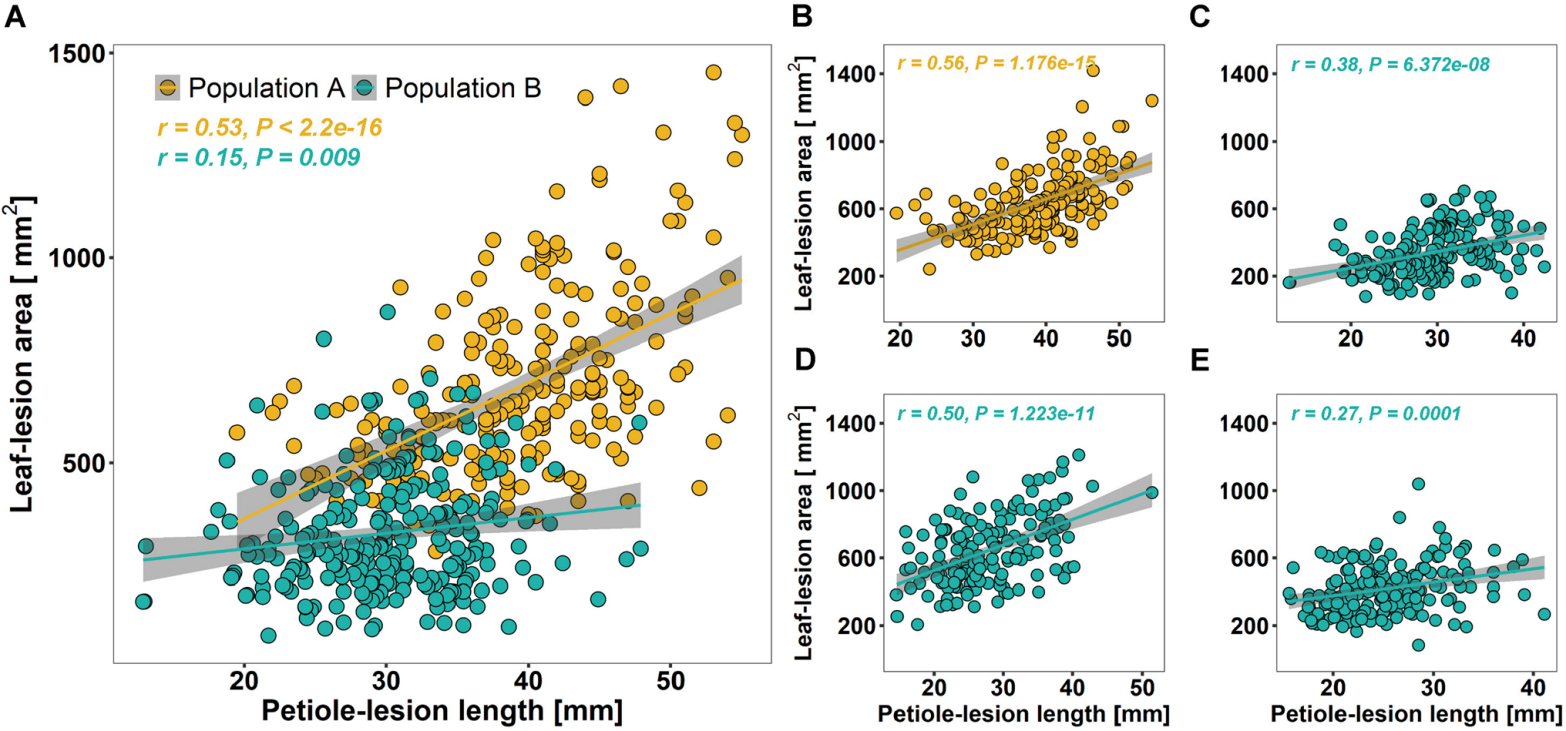
Scatterplots of leaf- and petiole-lesions in the two Brassica populations. **A** Lesion values of all *F*_2_ individuals in Population A (yellow) and Population B (cyan). **B** Lesion values of all genotyped *F*_2_ individuals in Population A. **C**–**E** Lesion values of all genotyped *F*_2_ individuals in Population B in all three resistance screenings. **C** First assay, **D** second assay, and **E** third assay. Correlations were calculated with Pearson’s correlation analysis. *r* coefficient of correlation, *P* value

Next, we determined the correlation between leaf- and petiole-lesions in both populations and identified significant correlations explaining about 28% of variance (*r* = 0.53) in Population A and about 2% of variance (*r* = 0.15) in Population B (Fig. 3A). The lower correlation in Population B was obviously caused by the slower lesion development. However, re-evaluation of Sclerotinia-resistance with a subset of Population B confirmed the positive correlation with an explainable variance of 25% (*r* = 0.50) and 7% (*r* = 0.27) in the two replications, respectively (Fig. 3D, E). Consequently, we selected the 207 *F*_2_ plants that were classified by both leaf and petiole tests in conformance with each other and 164 *F*_2_ individuals that showed moderate variation from the three categories from the both populations for genotyping with the 15-k Brassica SNP-chip assay (TraitGenetics, unpublished). The correlation between leaf- and petiole-lesions with the selected *F*_2_ plants from the both populations increased to 31% in Population A (*r* = 0.56) and to 14% (*r* = 0.38) in Population B (Fig. 3B, C). Lesion size distributions of all genotyped *F*_2_ individuals are shown in Supplementary Figure S3.

### Construction of a high-density genetic map

Overall, 9333 (68%) and 9156 (66%) SNPs were successfully called in *B. oleracea* and *B. villosa*. Of these, 392 and 215 SNPs were unique to *B. oleracea* and *B. villosa*, respectively. Filtering for polymorphic SNPs between both Brassica species reduced the set of markers to 2806. We combined the genotypes of all *F*_2_ individuals from both populations for genetic map construction. After quality filtering and removing duplicate markers, a genetic map was constructed from 1118 SNP marker with data from 361 *F*_2_ individuals (Supplementary Table S2 and Data S5). The core markers were ordered into 10 linkage groups with a total length of 784.9 cM and an average distance of 0.7 cM between adjacent markers. Linkage groups were assigned to chromosomes of the *B. oleracea* TO1000 reference genome (Parkin et al. 2014) according to a BLAST + search (Altschul et al. 1990; Camacho et al. 2009) of the SNP marker sequences. Low coverage of markers corresponding to the *B. oleracea* reference chromosome C04 resulted in two separate linkage groups (C04a and C04b). Overall, genetic positions of SNP markers were concordantly with their assumed physical positions in the *B. oleracea* TO1000 reference genome (Supplementary Data S5).

### QTL mapping for Sclerotinia-resistance in the wild Brassica populations

**Fig. 4:**
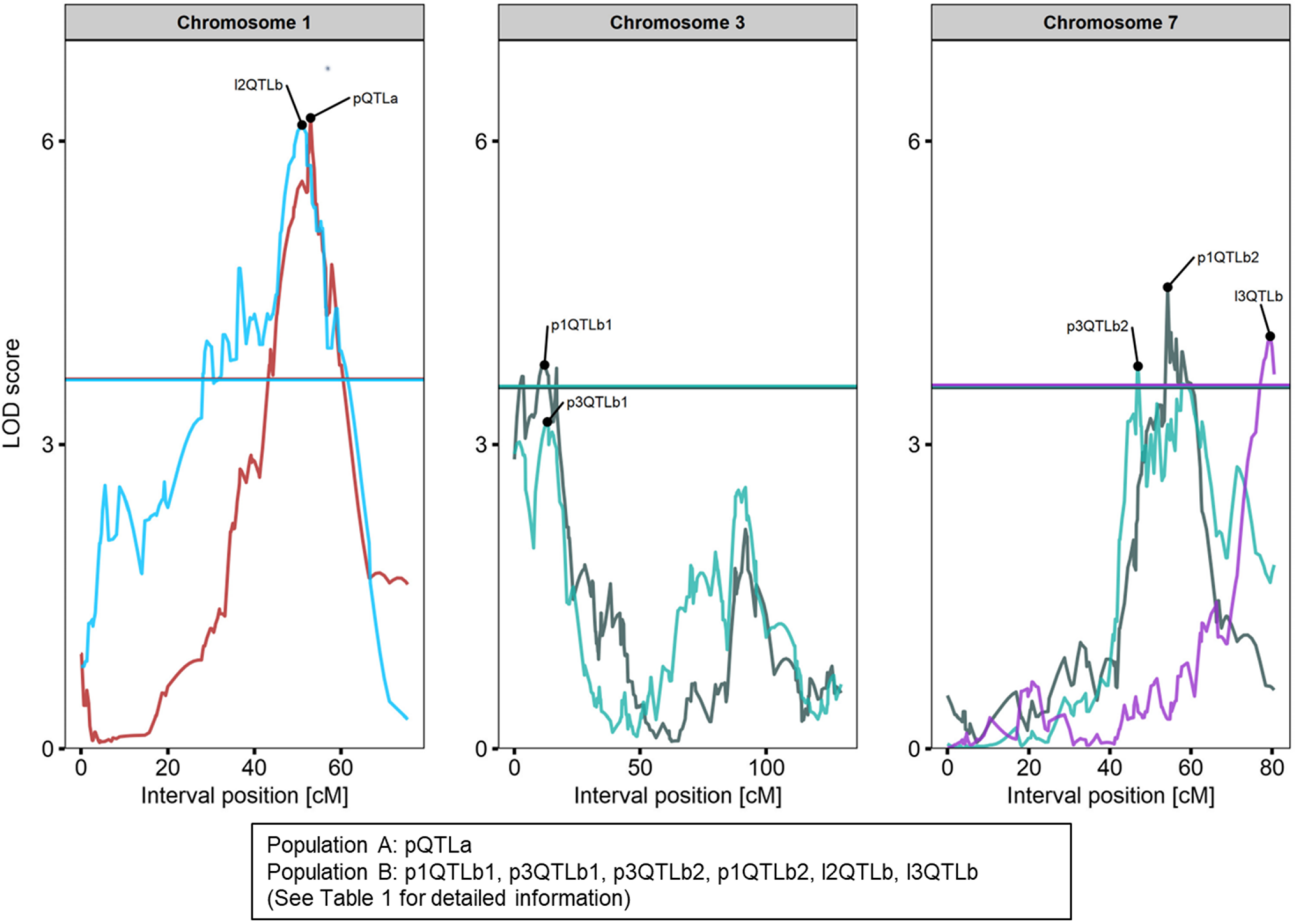
Logarithm of the odds (LOD) profiles of the identified QTL on chromosomes C01, C03, and C07 in the two *F*_2_ mapping populations. QTLs were labeled by trait (p = petiole, l = leaf) with numbers representing the replication and by mapping population (a = Population A, b = Population B) followed by a second number to distinguish multiple QTLs from one assay. LOD profiles from different assays are highlighted in different colors. Vertical lines indicate significance thresholds. Detailed information about each QTL are given in Table 1

In total, seven QTLs were identified in the two mapping populations (Fig. 4, Table 1). We identified one QTL (pQTLa) for petiole-resistance on linkage group C01 in Population A explaining 15.8% of the phenotypical variance (Table 1). No additional or interacting QTLs were detected in Population A. The QTL peak was detected at the marker Bn-scaff_19564_1-p17934 which was mapped to Scaffold01187 (~ 23 kb) of the *B. oleracea* TO1000 genome. Flanking markers were Bn-scaff_15749_1-p118178 (26,828,052 bp) and Bn-scaff_16929_1-p495739 (29,084,454 bp). The alleles of *B. villosa* in pQTLa reduced the petiole-lesions on average by 18% in comparison to *B. oleracea*. Six QTLs were additionally identified in Population B of which two QTLs (l2QTLb, l3QTLb) accounted for leaf resistance and four QTLs (p1QTLb1, p1QTLb2, p3QTLb1, p3QTLb2) for petiole-resistance (Table 1). In total, the QTLs for leaf- and petiole-resistance in Population B explained 26.9 and 26.6% of phenotypic variance, respectively. Two QTLs (p1QTLb1, p3QTLb2) on chromosome C03 and two QTLs (p1QTLb2, p3QTLb2) on chromosome C07 were repeatedly identified in Population B. The alleles of *B. villosa* reduced leaf-lesions by 7% and 35%, while petiole-lesions were reduced on average by 15%. Alleles from the susceptible *B. oleracea* were dominant in four of the six identified QTLs. All QTLs showed additive effects and no epistatic interactions were identified. The QTL for leaf resistance on chromosome C01 (l2QTLb2) explained 16.5% of phenotypic variance and overlapped with the QTL (pQTLa) for petiole-resistance from Population A. The peak was detected at Bn-scaff_22790_1-p152675 which corresponds to position 16,593,775 bp in the *B. oleracea* reference genome between the flanking markers Bn-scaff_15747_1-p105633 (14,270,425 bp) and Bn-scaff_22790_1-p1026422 (17,467,522 bp). The QTL for leaf resistance on chromosome C07 (l3QTLb) explained 10.4% of variance. The QTL peak was detected between the markers Bn-scaff_16110_1-p975852 (47,352,014 bp) and Bn-scaff_16110_1-p426547 (47,901,219 bp) corresponding to a 550 kbp region in the *B. oleracea* reference genome. The QTLs for petiole-resistance on chromosome C03 (p1QTLb1, p3QTLb1) explained 3.8% to 8.1% of variance. Both QTL peaks were detected between the markers Bn-scaff_16614_1-p734250 (2,054,448 bp) and Bn-scaff_16614_1-p174856 (3,106,932 bp). The QTLs for petiole-resistance on chromosome C07 (p1QTLb2, p3QTLb2) explained 4.8% to 9.9% of phenotypic variance. The QTL peaks were detected at Bn-scaff_16069_1-p2611780 (42,321,768 bp) and Bn-scaff_16069_1-p4306874 (44,016,862 bp) corresponding to a 1.7 mbp region in the *B. oleracea* reference genome. Logarithm of odds (LOD) profiles for all linkage groups are available in Supplementary Figure S4.

**Table 1:** Identified QTLs for Sclerotinia-resistance in the wild Brassica populations

Trait	QTL	LOD	LG	Position [cM]	Peak-marker^a^	*P* value	Var [%]	Add^b^	Dom^c^
Petiole	pQTLa	6.2	C01	53	Bn-scaff_19564_1-p17934	< 0.001	15.8	3.8	− 0.6
Leaf	l2QTLb	6.2	C01	51	Bn-scaff_22790_1-p152675	< 0.001	16.5	124	88.2
Leaf	l3QTLb	4.1	C07	79.6	Bn-scaff_16110_1-p976517	0.018	10.4	15.9	− 90.3
Petiole	p1QTLb1	3.8	C03	12	Bn-scaff_18936_1-p269153	0.032	8.1	2.0	1.3
Petiole	p1QTLb2	4.6	C07	54.3	Bn-scaff_16069_1-p4306874	< 0.01	9.9	2.4	1.1
Petiole	p3QTLb1	3.2	C03	13.1	Bn-scaff_18936_1-p269153	0.09	3.8	1.6	− 0.6
Petiole	p3QTLb2	3.8	C07	46.9	Bn-scaff_16069_1-p2611780	0.03	4.8	1.3	1.6

QTLs were labeled by trait (p = petiole, l = leaf) with numbers representing the replication and by mapping population (a = Population A, b = Population B) followed by a second number to distinguish multiple QTLs from one assay

*LOD* Logarithm of the odds, *LG* Linkage group, *cM* centiMorgan

^a^Marker at peak or nearest to the peak

^b^Additive effect. Positive value indicates that alleles from the susceptible parent (BRA1909) increase lesion values

^c^Dominant effect. Positive value indicates alleles from the susceptible parent (BRA1909) are dominant

### Comparative transcriptome analysis

**Fig. 5:**
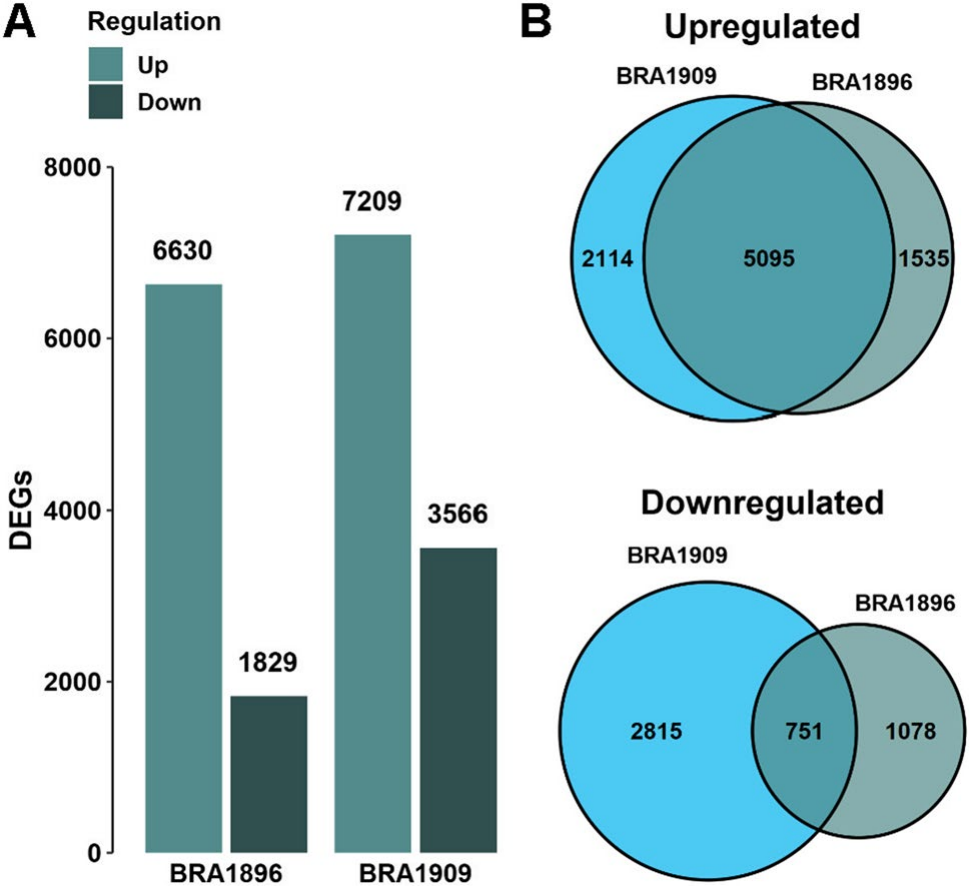
Differentially expressed gene (DEG) analysis between Sclerotinia- and mock-inoculated petioles of *B. villosa* (BRA1896) and *B. oleracea* (BRA1909). **A** Total amount of up- and downregulated DEGs in *B. villosa* and *B. oleracea*. **B** Comparison of up- and downregulated DEGs between the wild Brassica species. Genes were considered as differentially expressed in comparison to the mock-inoculated control based on an adjusted *P* value ≤ 0.05

To analyze plant transcriptional response to Sclerotinia infection, we applied RNAseq for a comparative transcriptome analysis on both *B. villosa* and *B. oleracea* at 8 hpi. Since the *B. villosa* genome is not yet available, we combined reference- and de novo-based RNAseq analyses to include genes that are not present in the *B. oleracea* TO1000 reference genome (presence/absence variants; PAVs). The alignment rate of RNAseq samples of mock-treated *B. oleracea* and *B. villosa* were on average 93.64% and 87.99%, respectively (Supplementary Table S3). In Sclerotinia-inoculated samples, the alignment rate of the sequences decreased to about 77.70% in *B. oleracea* and 74.59% *in B. villosa*, respectively. Hierarchical clustering and PCA showed that RNAseq samples grouped accordingly to species and treatments (Supplementary Figure S5). Almost all variance (97%) between the samples was explained by the factors species (58%) and treatment (39%). Overall, 63,995 expressed genes were identified in the wild Brassica species of which 15,251 transcripts (putative PAVs) could not be aligned to the *B. oleracea* reference genome. In total, 8,459 DEGs were identified in the resistant and 10,775 DEGs in the susceptible species, respectively (Fig. 5A), from which 5095 up- and 751 downregulated DEGs were common in both species (Fig. 5B).

Transcriptomic data suggested that the Sclerotinia infection induced an early transcriptional response in both species, which were associated with genes involved in ‘defense response,’ ‘response to chitin,’ ‘immune response,’ and ‘response to decreased oxygen levels,' as indicated by GO enrichment analysis. In total, 111 biological processes were commonly enriched in the resistant and the susceptible species, of which the ‘response to chitin’ (BRA1896: *P* adj. = 2.31e−60; BRA1909: *P* adj. = 3.59e−38) represented the most enriched process in both species. Further on, the comparison of the DEGs associated with this biological process identified 130 DEGs common to both species and 39 DEGs specific for the resistant *B. villosa*. The resistance-specific DEGs included homologs of WRKY, NAC, and zinc finger transcription factors (TFs). For instance, a homolog (Unigene.4449) of the zinc finger protein ZAT12 showed a 5.3-fold (log_2_-fold: 2.41) induction in *B. villosa* but no significant induction (0.38-fold; *P* adj. = 0.73) in *B. oleracea*. *ZAT12* is a key component in oxidative stress response signaling in *A. thaliana* (Rizhsky et al. 2004). In addition, significant responses of ET-, abscisic acid (ABA)-, salicylic acid (SA)-, and jasmonic acid (JA)-activated pathways were observed in both species. While phytoalexin-related processes were highly enriched in *B. villosa*, the secondary metabolic processes identified in *B. oleracea* were mainly associated with glucosinolate metabolism.

### Pathways and genes specifically enriched in *B. villosa*

Diverse biological processes were identified to be specifically activated in *B. villosa*. The processes were among others enriched with DEGs that are associated with ‘positive regulation of defense response,’ ‘negative regulation of cell death,’ and ‘response to reactive oxygen species.’ From them, 25 genes were specifically induced in the resistant *B. villosa*, including several homologs of ET responsive factors, receptor-like proteins (RLPs), and RLKs associated with the perception of necrosis-inducing proteins (Supplementary Table S4). Of these, seven de novo genes (genes with a ‘Trinity_’ prefix) were not found in the *B. oleracea* TO1000 reference genome and not expressed (zero read count) in samples of the susceptible *B. oleracea*. Interestingly, one homolog (Unigene.13474) of the multifunctional RLK *BOTRYTIS INDUCED KINASE 1* (*BIK1*) was specifically induced in *B. villosa* with 1.9-fold (log_2_-fold: 0.91) in response to the Sclerotinia-inoculation. We also identified one homolog (Trinity_DN2466_c0_g1_i7) of the *RECEPTOR-LIKE PROTEIN 30* (*RLP30*) to be specifically expressed 2.63-fold (log_2_-fold: 1.4) in the resistant *B. villosa*. *RLP30* was shown to mediate enhanced resistance to necrotrophic pathogens such as *S. sclerotiorum* and *B. cinerea* in *A. thaliana* (Zhang et al. 2013). In total, we identified 413 de novo DEGs significantly induced in *B. villosa*. Of these 58 are functionally associated with the plant defense response of which 34 were not detectable (zero read count) in *B. oleracea* (Table 2). As revealed by in silico sequence analysis, these genes encode among others for putative RLPs, chitinases, disease resistance proteins, zinc finger family proteins, ET response factors (ERFs), and WRKY TFs.

**Table 2:** De novo assembled genes specifically expressed in *B. villosa* (BRA1896) and associated with plant defense response

Gene_ID	Log_2_-fold change		Homolog in *A. thaliana*	Putative annotation
	BRA1896	BRA1909		
Trinity_DN1159_c1_g1_i10	**1.68**	0.00	AT1G11310	Transmembrane domain protein
Trinity_DN2585_c0_g1_i2	**2.68**	0.00	AT2G33580	Receptor-like kinase
Trinity_DN1605_c0_g2_i5	**3.24**	0.00	AT2G38470	WRKY-transcription factor
Trinity_DN2365_c0_g1_i5	**1.22**	0.29	AT5G28040	Leucine zipper transcription factor
Trinity_DN46357_c0_g1_i1	**2.20**	2.35	AT1G70130	Protein kinase
Trinity_DN628_c1_g1_i3	**1.34**	0.00	AT5G18370	Leucine-rich repeat domain receptor
Trinity_DN1154_c0_g1_i3	**1.73**	0.00	AT1G61390	Protein kinase
Trinity_DN557_c0_g1_i10	**2.22**	3.81	AT1G15520	ABC-transporter
Trinity_DN5013_c0_g1_i25	**2.52**	0.00	AT3G09830	Receptor-like kinase
Trinity_DN7883_c0_g1_i17	**1.68**	0.00	AT1G74360	Serine/threonine kinase
Trinity_DN5417_c0_g1_i33	**2.81**	0.00	AT5G27420	Ubiquitin ligase
Trinity_DN4012_c0_g1_i4	**4.94**	0.00	AT4G23310	Cysteine-rich receptor-like protein kinase
Trinity_DN21071_c0_g2_i2	**5.05**	2.23	AT2G02220	Receptor-like protein
Trinity_DN1154_c0_g1_i46	**3.11**	0.00	AT1G61390	Protein kinase
Trinity_DN2466_c0_g1_i7	**1.40**	0.00	AT3G05360	Receptor-like protein
Trinity_DN3122_c1_g3_i4	**1.43**	0.00	AT2G43590	Chitinase protein
Trinity_DN242_c0_g1_i19	**1.30**	0.00	AT1G10170	Transcription repressor
Trinity_DN4251_c0_g1_i18	**5.13**	0.50	AT1G02305	Capase
Trinity_DN7129_c0_g1_i1	**1.89**	0.00	AT4G02420	Protein kinase
Trinity_DN651_c3_g2_i2	**2.48**	0.00	AT4G17490	Ethylene response factor
Trinity_DN1154_c0_g1_i11	**3.06**	0.00	AT1G61380	Receptor-like kinase
Trinity_DN12674_c0_g2_i4	**7.19**	0.00	AT2G40140	Zinc finger family protein
Trinity_DN1095_c0_g1_i27	**1.60**	− 0.47	AT1G19180	Nuclear-localized protein
Trinity_DN1554_c0_g1_i16	**2.55**	1.87	AT2G37940	Inositol phosphorylceramide synthase
Trinity_DN3122_c1_g3_i1	**1.81**	0.49	AT2G43590	Chitinase protein
Trinity_DN6286_c0_g1_i18	**3.44**	4.00	AT3G04720	Chitin-binding protein
Trinity_DN8160_c0_g1_i1	**1.02**	− 0.54	AT2G37040	Phenylalanine ammonia-lyase
Trinity_DN1605_c0_g2_i6	**3.01**	− 2.37	AT2G38470	WRKY-transcription factor
Trinity_DN770_c0_g1_i6	**2.73**	0.00	AT2G03760	Sulfotransferase
Trinity_DN4012_c0_g1_i2	**1.94**	− 3.39	AT4G23310	Cysteine-rich receptor-like protein kinase
Trinity_DN624_c0_g1_i2	**1.24**	0.00	AT5G03320	Protein kinase
Trinity_DN1627_c0_g2_i4	**2.34**	0.00	AT2G32240	PAMP-induced protein
Trinity_DN787_c0_g3_i1	**4.25**	0.00	AT2G32680	Receptor-like protein
Trinity_DN882_c0_g2_i3	**1.46**	0.00	AT2G37040	Phenylalanine ammonia-lyase
Trinity_DN10838_c0_g1_i8	**1.62**	1.45	AT4G34131	UDP-glucosyl transferase
Trinity_DN159_c0_g3_i18	**3.00**	− 0.89	AT2G21660	Glycine-rich RNA binding protein
Trinity_DN590_c0_g1_i10	**2.36**	0.00	AT3G05200	Putative ring-h2 zinc finger protein
Trinity_DN2969_c0_g1_i1	**3.51**	0.00	AT1G80840	WRKY-transcription factor
Trinity_DN5502_c0_g1_i19	**1.13**	− 0.47	AT4G25030	Plastid localized protein
Trinity_DN871_c0_g1_i3	**1.79**	0.00	AT5G64120	Peroxidase
Trinity_DN3122_c1_g3_i2	**1.57**	1.45	AT2G43590	Chitinase protein
Trinity_DN1154_c0_g1_i24	**2.59**	0.00	AT1G61390	Protein kinase
Trinity_DN4012_c0_g3_i1	**2.34**	0.43	AT4G23180	Receptor-like protein
Trinity_DN839_c0_g1_i16	**2.61**	0.00	AT5G06320	Disease resistance gene
Trinity_DN1154_c0_g1_i51	**1.92**	0.00	AT1G61380	Receptor-like kinase
Trinity_DN1607_c1_g4_i1	**1.41**	0.00	AT5G47220	Ethylene response factor
Trinity_DN787_c0_g3_i2	**2.24**	0.00	AT2G32680	Receptor-like protein
Trinity_DN1900_c2_g1_i4	**1.22**	0.66	AT5G48380	Receptor-like kinase
Trinity_DN1607_c1_g6_i1	**3.30**	− 2.70	AT2G44840	Ethylene response factor
Trinity_DN1095_c0_g1_i8	**1.02**	0.00	AT1G19180	Nuclear-localized protein
Trinity_DN686_c0_g1_i20	**1.64**	0.88	AT1G80820	Cinnamoyl CoA-reductase
Trinity_DN7883_c0_g1_i10	**2.06**	0.00	AT1G74360	Serine/threonine kinase
Trinity_DN9783_c0_g1_i3	**1.62**	− 0.08	AT1G58602	Disease resistance protein
Trinity_DN1605_c0_g3_i6	**3.52**	− 0.47	AT2G38470	WRKY-transcription factor
Trinity_DN21071_c0_g2_i1	**1.84**	0.45	AT2G02220	Receptor-like protein
Trinity_DN1154_c0_g1_i45	**1.79**	0.00	AT1G61390	Protein kinase
Trinity_DN1795_c0_g2_i9	**1.61**	0.00	AT3G09980	Microtubules-associated protein
Trinity_DN1605_c0_g3_i1	**5.41**	− 0.47	AT2G38470	WRKY-transcription factor

Significant log_2_-fold change is marked in bold (*P* adj. ≤ 0.05). BRA1909 = *B. oleracea*

### Enhanced ET-mediated signaling in *B. villosa*

Genes with significant differences in their expression patterns between the both species were denoted as interaction DEGs (iDEGs) for further analysis. From 854 iDEGs, 542 were significantly induced in *B. villosa*, while 312 were stronger induced in *B. oleracea*. Strikingly, the GO enrichment analysis with the iDEGs showed that most of these genes were among others associated with ‘response to chitin,’ ‘defense response,’ ‘hormone-mediated signaling pathway,’ ‘immune system process,’ and ‘ET-activated signaling pathway.’ In particular, 23 ET–related iDEGs noticeably differed between the both species (Supplementary Table S5). Most of these iDEGs encode putative ERFs and were stronger induced in *B. villosa.* For example, a homolog of *ETHYLENE RESPONSE FACTOR 1* (*ERF1*; Unigene.2457) was found to be significantly induced in *B. villosa* (11.7-fold) compared with *B. oleracea* (1.3-fold). By contrast, ABA-related genes were overall stronger induced in *B. oleracea*. For example, a homolog (Unigene.6344) of the E3 ubiquitin–protein ligase *RHA2B* was 5.1-fold (log_2_-fold: 2.35, *P* adj. = 0) induced in *B. oleracea*, while no significant induction (log_2_-fold: 0.45, *P* adj. = 0.56) was observed in *B. villosa* (Data S7). *RHA2B* is involved in the positive regulation of ABA-mediated signaling (Li et al. 2011).

**Fig. 6:**
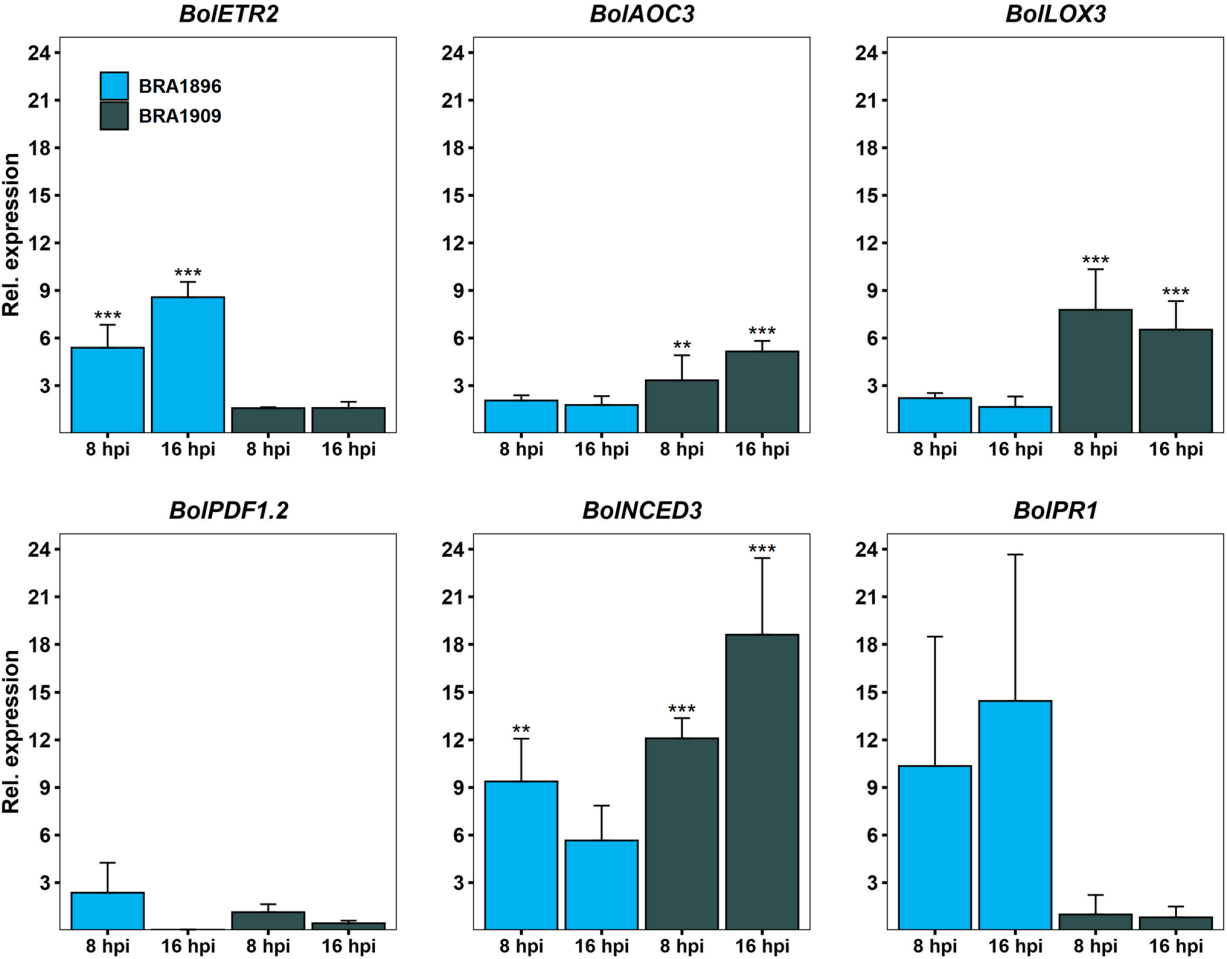
Marker gene expression analysis by reverse-transcribed quantitative PCR (RT-qPCR) in petioles of *B. villosa* (BRA1896, blue) and *B. oleracea* (BRA1909, gray) at 8 hpi to 16 hpi. Asterisks indicate a significant induction compared to the control group calculated by a linear model using generalized least squares and multiple contrast tests. **P adj. < 0.01, ***P adj. < 0.001. Error bars represent standard deviation of at least two biological replications. Primer used for RT-qPCR are listed in Supplementary Table S1 (colour figure online)

To monitor changes in the hormone-mediated pathways, transcriptional profiling of known marker genes was conducted by RT-qPCR for both species at 8 hpi and 16 hpi. They include *ALLENE OXIDASE CYCLASE 3* (*AOC3*; Bo9g075870) and *LIPOXYGENASE 3* (*LOX3*; Bo8g067210) for JA; *ETR2* (Unigene.2465) for ET; *PLANT DEFENSIN 1.2* (*PDF1.2*; Bo2g086460) for the JA/ET branch; *NINE-CIS-EPOXYCAROTENOID DIOXYGENASE 3* (*NCED3*; Bo5g130280) for ABA and *PATHOGENESIS-RELATED GENE 1* (*PR1*; Bo3g088360) for SA (Fig. 6). As expected, RT-qPCR data at 8 hpi were overall in accordance with the RNAseq data (Supplementary Figure S6). Thereby the expression of *BolETR2* (Unigene.2465) increased from 5.39-fold at 8 hpi to 8.57-fold at 16 hpi in the resistant *B. villosa*, while only about 1.5-fold induction occurred in the susceptible *B. oleracea* at both time points. In contrast, the JA marker genes *BolAOC3* (Bo9g075870) and *BolLOX3* (Bo8g067210) were induced up to 7.8-fold in the susceptible *B. oleracea* at 8 hpi and 16 hpi, whereas their expression in *B. villosa* increased about 2-fold on average. Unexpectedly, no significant change in *BolPDF1.2* (Bo2g086460) was observed in both Brassica species. The expression level of *BolNCED3* (Bo5g130280) was higher in *B. oleracea* than in *B. villosa* and increased from 12.1-fold to 18.6-fold in *B. oleracea* and decreased from 9.38-fold to 5.66-fold in *B. villosa,* respectively. Also, *BolPR1* (Bo3g088360) expression patterns varied between *B. villosa* (10.4-fold at 8 hpi and 14.5-fold at 16 hpi) and *B. oleracea* (0.99-fold at 8 hpi to 0.81-fold at 16 hpi). Taken together, RT-qPCR data support the differences in the ET-and ABA-mediated signaling pathways between *B. villosa* and *B. oleracea* as observed from the RNAseq data.

### Identification of a RLK gene cluster in the QTL on chromosome C07

To identify genes that may contribute to Sclerotinia-resistance in *B. villosa*, we surveyed the genomic regions of the QTLs identified in the *B. oleracea* TO1000 reference genome with our RNAseq data. As a result, 36 genes were found to reside in the QTLs which showed a stronger expression in the resistant *B. villosa* and are functionally related to plant defense response. They include putative disease resistance proteins (e.g., Unigene.2315, Unigene.27237), MYB TFs (Unigene.7579), ET receptors (Unigene.2258), and RLKs (Unigene.27214; Table 3). Moreover, we identified a cluster of five putative cysteine-rich repeat RLKs on chromosome C07, which were significantly and/or more strongly induced in the resistant *B. villosa* in responsive to the Sclerotinia infection. Adjacent to the five RLKs, a homolog (Unigene.27237) of a toll-interleukin-resistance (TIR) domain protein and a homolog (Bo7g109370) of a thaumatin-like protein were identified as well, which showed a 4.79- and 4.92-fold increased expression in resistant petioles, respectively.

**Table 3:** Pathogenesis-related (PR) genes in the identified QTLs in the *B. oleracea* TO1000 reference genome

Chr	Position [bp]	Gene_ID	Log_2_-fold change		Homolog in *A. thaliana*	Putative annotation
			BRA1896	BRA1909		
pQTLa						
C1	27,090,180–27,094,365	Unigene.2258	0.56	− 0.27	AT1G60060	ET receptor
C1	27,961,418–27,964,840	Unigene.2305	0.28	− 0.05	AT5G09890	Protein kinase
C1	28,166,345–28,169,898	Unigene.2315	**1.23**	− 0.14	AT1G61100	Disease resistance protein
C1	28,665,765–28,668,996	Unigene.2328	**1.37**	0.28	AT1G61380	Receptor kinase
C1	28,684,815–28,689,499	Unigene.2332	**0.82**	0.20	AT1G61490	Protein kinase
C1	28,775,666–28,778,647	Bo1g098240	1.56	− 0.30	AT1G61590	Protein kinase
*l2QTLb*						
C1	14,675,631–14,677,152	Unigene.1621	1.52	− 0.68	AT4G32480	Sugar phosphate exchanger
C1	15,950,476–15,952,442	Unigene.1707	2.41	0.64	AT4G16260	Beta-1,3-endoglucanase
C1	16,945,305–16,951,076	Bo1g057070	3.77	0.15	AT5G38340	Disease resistance protein
C1	17,209,332–17,213,228	Bo1g057280	1.25	0.02	AT4G14940	Copper amine oxidase
*l3QTLb*						
C7	47,411,341–47,413,075	Unigene.27801	0.80	0.03	AT4G37150	Methyl salicylate esterase
C7	47,471,969–47,474,259	Unigene.27814	**1.13**	**0.79**	AT4G36900	ERF/AP2 transcription factor
C7	47,507,341–47,508,305	Unigene.27815	**1.61**	**2.67**	AT4G37290	PAMP-induced peptide
C7	47,591,970–47,593,598	Unigene.27828	0.42	− 0.07	AT4G37610	TAZ-domain protein
C7	47,645,847–47,647,163	Bo7g118680	2.05	− 1.10	AT4G37690	Glycosyltransferase
*p1QTLb1/p3QTLb1*						
C3	2,464,575–2,468,509	Unigene.7573	0.86	− 0.92	AT5G11250	TIR-NBS-LRR protein
C3	2,505,710–2,510,061	Unigene.7579	0.33	− 1.22	AT5G11510	MYB-transcription factor
C3	2,590,387–2,591,964	Unigene.7588	2.17	0.76	AT1G05890	RING/U-box superfamily protein
C3	2,678,919–2,679,799	Unigene.7604	**2.12**	0.21	NA	NA
C3	2,925,738–2,929,237	Bo3g008890	2.86	− 2.40	AT5G12920	WD40 repeat-like superfamily protein
*p2QTLb2/p3QTLb2*						
C7	42,428,839–42,430,131	Bo7g108060	1.22	− 0.33	AT4G22680	MYB-domain protein
C7	42,449,575–42,451,152	Unigene.27187	0.63	0.00	AT4G22690	Cytochrome P450 protein
C7	42,731,387–42,734,266	Unigene.27214	**1.16**	− 0.57	AT4G23130	Cysteine-rich receptor-like kinase
C7	42,737,429–42,739,520	Unigene.27215	3.29	0.94	AT4G23130	Cysteine-rich receptor-like kinase
C7	42,744,618–42,745,142	Unigene.27216	**4.72**	2.52	AT4G23130	Cysteine-rich receptor-like kinase
C7	42,793,701–42,796,988	Unigene.27221	**1.18**	0.49	AT4G23230	Cysteine-rich receptor-like kinase
C7	42,800,478–42,803,336	Unigene.27222	**2.39**	0.42	AT4G23240	Cysteine-rich receptor-like kinase
C7	42,926,260–42,931,088	Unigene.27237	2.26	0.51	AT4G23515	TIR-domain protein
C7	42,967,394–42,969,656	Unigene.27241	**0.99**	− 0.01	AT4G23570	Protein SGT1 homolog A
C7	43,082,687–43,083,634	Unigene.27253	3.96	0.79	AT4G23720	Transmembrane protein (DUF119)
C7	43,297,480–43,298,989	Bo7g109370	2.30	− 1.88	AT4G24180	Thaumatin-like protein
C7	43,317,954–43,319,839	Unigene.27284	0.82	0.06	AT4G24240	Calmodulin-binding protein
C7	43,411,597–43,412,228	Unigene.27297	2.74	− 0.03	AT4G30400	RING/U-box superfamily protein
C7	43,515,228–43,516,110	Unigene.27316	**2.50**	**1.12**	AT4G24960	HVA22-like protein
C7	43,568,299–43,569,252	Unigene.27323	2.08	− 1.18	AT4G25130	Methionine sulfoxide reductase
C7	43,865,115–43,870,847	Bo7g110600	2.22	− 1.36	AT4G25960	*P*-glycoprotein

## Discussion

### *B. villosa* is a promising source of genetic resistance against Sclerotinia

Mei et al. (2011) and Taylor et al. (2018) reported wild *B. incana* to be highly resistant to Sclerotinia. Here, we demonstrate for the first time that a wild *B. villosa* accession (BRA1896) represents a novel and more promising genetic source for breeding Sclerotinia-resistance in oilseed rape. The partial transfer of the Sclerotinia-resistance of the wild *B. incana* into the *B. napus* has been demonstrated by Mei et al. (2020) leading to improvement of Sclerotinia-resistance to an average of 35% as compared with the partially resistant Zhongshuang 9. The QTLs from *B. incana* explained an average effect of ca. 11% of phenotypical variance (Mei et al. 2020). Although the QTLs identified from *B. villosa* showed a similar effect magnitude but with a higher level of quantitative resistance, it is reasonable to believe that the introgression of them into the *B. napus* will be worth the effort for breeding Sclerotinia-resistant oilseed rape.

### The petiole-assay is an efficient and reliable method to assess Sclerotinia-resistance in wild Brassica species

Leaf- and petiole-inoculation assays were both applied for this study. We observed that a fast drying of PDA plugs, which were used as medium for Sclerotinia, on the leaf-surfaces and irregularities of the leaf-surfaces severely impeded the inoculation process of the leaf assay in some cases. In the petiole-assay, PDA-plugs were well protected from dehydration by the surrounding pipette tip and the fungus could easily infiltrate the petiole via the open cut of the petiole tissue. This may explain the discrepancy of our phenotypic data obtained from two populations: A decelerated symptom development in the leaf assay and smaller leaf-lesion sizes in Population B than in Population A. However, the petiole-assays showed lesion sizes in a similar range for both populations, thus be able to discriminate the resistant and susceptible parent species with high significance (*P* values < 0.01, Supplementary Figure S7). This may explain why the detached petiole-assays yielded more stable QTLs than the leaf assay in this study. Though the inoculation of leaves is a well-established Sclerotinia-inoculation technique (Joshi et al. 2016; Mei et al. 2011; Zhao and Meng 2003), our data suggest that the detached petiole-assay is a more efficient and reliable screening method, especially when the stem-inoculation is not possible as in our case.

There are contradictory reports in regarding the correlation between leaf-, stem-, and petiole-resistance. Taylor et al. (2018) found no significant correlation between leaf- and petiole-resistance results by analyzing a set of wild Brassica species, whereas it was given between petiole- and stem-resistance in a collection of *B. napus* accessions. Uloth et al. (2013) and You et al. (2016) found no association between leaf- and stem-resistance under field conditions after investigation on diverse Brassica species. But, a correlation between leaf- and stem-resistance was reported by Mei et al. (2011, 2013) even under field as well as controlled environments by artificial inoculation. In this study, we demonstrate a significant correlation (*P* values > 0.01) between leaf- and petiole-resistance across all assays. A rather lower explainable variance, ranging from 2 to 31%, might be mainly attributed to the technical sensitivity of the leaf assay, which masked the correlation analysis. It should be noted that the results of these two assays may reflect different resistance mechanisms: The leaf assay includes the additional effect from preformed resistance, a physical barrier to the fungus in general, while the petiole-assay mainly reflects the plant defense response induced by the fungal infection. Thus, the combination of leaf- and petiole-assays as did in this study may facilitate the screening and identifying of effective Sclerotinia-resistance. Further studies, e.g., by the stem-inoculation under natural conditions, are needed to substantiate the functional significance of the QTLs identified in this study.

### Diverse Sclerotinia-resistance mechanisms existing in different wild Brassica species

Seven QTLs for Sclerotinia-resistance in *B. villosa* were identified, from which two were from leaf- and five from the petiole-assays. Strikingly, the major QTL identified from the petiole-assay (pQTLa) in Population A is overlapping with the major QTL for leaf resistance (l2QTLb) in Population B, with both accounting for approx. 16% of phenotypic variance, respectively. This finding suggests that the underlying resistance mechanism may be relying on common genetic resistance factors. We also detected two partially overlapping QTLs from the petiole-assay (p1QTLb2, p3QTLb2) and one QTL from the leaf assay (l3QTLb) on chromosome C07, but due to their genetic and physical distance, these QTLs do not appear to be directly connected to each other. Mei et al. (2013) analyzed one *F*_2_ mapping population from a cross between wild *B. incana* (resistant) and the cultivated *B. oleracea* var. *alboglabra* (susceptible). They detected a major QTL for Sclerotinia-resistance on chromosome C09 explaining up to 28.4% of phenotypical variance as well as minor QTLs on chromosomes C01, C03, C04, and C07. But, the use of different markers from various references as well as a different *B. oleracea* reference genome (JZS; *B. oleracea* sp. *capitata*) makes a direct comparison with our results not possible. Nevertheless, we compared our results with those reported by Li et al. (2015) who had physically integrated QTLs for Sclerotinia-resistance from several studies including those reported by Mei et al. (2013) to the *B. napus* genome. Conspicuously the QTLs on chromosome C01 from our study are physically overlapped with those reported by Mei et al. (2013), unfortunately there is no overlapping QTL on chromosome C07, where most *B. villosa* QTLs reside. Recently, SNPs significantly associated with SSR-resistance were detected on chromosome C03 in *B. napus* (Shahoveisi et al. 2021; Roy et al. 2021). Comparing the physical positions reported in the *B. napus* genome with those of the reference genome revealed that one SNP at position 7.893,201 bp in *B. napus* (Roy et al. 2021) is corresponding to 3.334.677 bp in the *B. oleracea* genome which is close to the QTL marker Bn-scaff_16614_1-p174856 (3,106,932 bp) in this study.

It is to note that the QTLs on chromosome C01 in our mapping populations contributed to phenotypic variance of about 16%, while only 8.4% of phenotypic variance was reported by Mei et al. (2013). These data suggest that there exist distinct Sclerotinia-resistance mechanisms in different wild Brassica species, but some genetic factors being involved in resistance seem to be conserved, thus supporting that the *B. oleracea* complex represents a valuable genetic source for breeding Sclerotinia-resistance in oilseed rape.

### Sclerotinia-resistance in *B. villosa* is linked to the ET-activated signaling pathway

The enriched biological processes and pathways identified in this study differ from those in *B. incana* (Ding et al. 2019). The resistance in *B. incana* is mainly associated with an enhanced cell wall integrity and an accelerated Ca^2+^ signaling which, as the authors suggested, regulates the production of an early respiratory burst via the accumulation of reactive oxygen species (ROS; Ding et al. 2019). Furthermore, they demonstrated that *S. sclerotiorum* takes over control of the copper ion homeostasis in susceptible host genotypes to scavenge and detoxify plant ROS to repress the oxidative burst in the early stage of infection, while resistant host genotypes such as *B. incana* were less disturbed in their copper ion homeostasis (Ding et al. 2020). However, the reported mechanisms are not linked to the previously identified QTLs in *B. incana*. Thus, the main genetic factors and mechanisms governing the resistance in this species remain unsolved. Yet, we did not find any evidence for the involvement of the copper ion homeostasis in *B. villosa*, but identified processes related to the perception of ROS, the regulation of cell death, as well as an enhanced immune and defense response. Our data, however, underpin the role of initiation of an early respiratory burst by the fungal infection in plant resistance to *S. sclerotiorum. S*everal related genes showed contrasting expression patterns in resistant and susceptible species, including, e.g., Unigene.4449, a homolog of the key gene ZAT12 in oxidative stress response signaling in *A. thaliana* (Rizhsky et al. 2004) that was 5.3-fold induced in *B. villosa* but not significantly induced in *B. oleracea.*

The early respiratory burst was apparently differently regulated in the resistant and susceptible species. In *B. villosa*, it was found to be associated with an enhanced expression of genes related to the ET-activated signaling pathway, whereas the JA-mediated signaling pathway was less activated. This may be a result of the interference of JA- and SA-pathways as, in support of this, a highly elevated transcript abundance of *PR1* was concurrently observed in *B. villosa* as compared to *B. oleracea.* As confirmed by RT-qPCR, the expression level of *ETR2,* an ET-marker gene, significantly increased from 8 to 16 hpi only in *B. villosa*, but not in the susceptible *B. oleracea,* whereas two JA-marker genes *AOC3* and *LOX3* were in contrast induced only in *B. oleracea* but not in *B. villosa* at both time points, respectively*.*

The ET- and JA-mediated signaling pathways are key components in regulating plant defense to necrotrophic pathogens by synergizing the ERF branch via *ERF1*/*ORA59* (Broekgaarden et al. 2015; Pré et al. 2008). A key marker gene of the ERF branch is *PDF1.2* that is regulated by *ORA59*, an essential integrator of the ET- and JA-signal transduction pathway (Pré et al. 2008). Interestingly, our RNAseq data revealed a significant elevation of the expression level of one *ORA59*-like gene (Bo8g114710) in *B. villosa* but without induction of *PDF1.2* in both Brassica species. Thus, we speculate that the ET- and JA-signaling pathways do not act through the common ERF branch but trigger different immune responses in the two Brassica species. An early oxidative burst in *B. villosa* could be a result of the ET-activated signaling. To clarify this, more experiments are needed.

Yang et al. (2017) reported that infection of resistant rice cultivars with the blast fungus *Magnaporthe oryzae* also induced the ET-mediated signaling pathway that increased ROS accumulation and the production of phytoalexins. An significant enrichment of DEGs associated with the phytoalexin metabolism was also identified in *B. villosa,* thus suggesting a signaling network similar to that described in rice (Yang et al. 2017). In the susceptible *B. oleracea*, we found that the glucosinolate and sulfur compound metabolic processes were enhanced after Sclerotinia infection, in line with the observation in *B. napus* as a result of an enhanced JA-mediated signaling (Wei et al. 2016). In addition, we noticed the activation of 19 iDEGs involved in the ABA-signaling pathway in *B. oleracea*. Interestingly, the orthologue Bo2g159220 of the MYC branch marker gene *VSP2* was neither significantly induced in *B. oleracea* nor in *B. villosa*. The JA-mediated MYC branch, antagonistic to the ERF branch, proved to be responsible for defense against herbivores and co-regulated by ABA (Broekgaarden et al. 2015; Vos et al. 2015). These data suggest a complex interplay of hormone-mediated signalings occurs during resistance to Sclerotinia in *B. villosa*.

### Possible candidate genes for Sclerotinia-resistance

A large set of candidate genes were identified in the resistant *B. villosa,* which are functionally associated with the immune response or PR genes and linked to our QTLs. Notably, a small cluster of RLKs together with two putative disease resistance proteins reside in the QTL on chromosome C07. Most of the RLK genes were induced specifically in the resistant *B. villosa*. Two putative disease resistance genes were also found in the resistant *B. villosa* in the QTLs on chromosome C01. Further analyses are needed to clarify whether these genes are involved in Sclerotinia-resistance. Since the reference-based analysis relies on the *B. oleracea* reference genome, we integrated the de novo transcriptome assembler Trinity (Grabherr et al. 2011) into our RNAseq analysis to identify putative PAVs specific for *B. villosa* but not present in the susceptible *B. oleracea*. This approach let to identification of additional 34 putative PAVs associated with the plant defense response in *B. villosa*. Because alignment with the reference genome failed, no genetic linkage could be established with the identified QTLs. Thus, re-sequencing of the *B. villosa* genome will shed more light on the role of these genes in Sclerotinia-resistance and facilitate the identification of candidate genes for breeding Sclerotinia-resistant oilseed rape in the future.

## Conclusion

By QTL mapping and transcriptome analysis, we demonstrate for the first time that the wild accession *B. villosa* is a novel and valuable genetic source of quantitative resistance against the fungal pathogen *S. sclerotiorum*. The ET-activated signaling may represent a key signaling pathway to the activation of plant *S. sclerotiorum* defense response, associated with an early ROS production and an increased production of phytoalexins in the resistant *B. villosa*. Moreover, the genes and the QTLs identified in this study are promising candidates for investigation on molecular plant-*S. sclerotiorum* interactions as well as for breeding of resistant oilseed rape varieties against *S. sclerotiorum* infection.

## Supplementary Information

Below is the link to the electronic supplementary material.

Supplementary file1 (XLSX 12204 KB)

Supplementary file2 (PDF 3301 KB)

Supplementary file3 (PDF 123 KB)

## Data Availability

The main data is provided in the electronic supplementary material. Additional data and pre-calculated permutations for the QTL analysis are available on request. Raw sequencing data is available at the NCBI Sequence Read Archive (PRJNA706136).

## Code availability

The main code is available at https://zenodo.org/record/5524469.
