# Supplementary material for: QTL mapping and transcriptome analysis identify novel QTLs and candidate genes in Brassica villosa for quantitative resistance against Sclerotinia sclerotiorum
Source: Theor Appl Genet. 2023 Mar 26;136(4):86. doi: 10.1007/s00122-023-04335-9 (PMC10040396; doi:10.1007/s00122-023-04335-9)
Supplement: Supplementary file 2 — Supplementary file2 (PDF 3301 KB) [file 122_2023_4335_MOESM2_ESM.pdf]

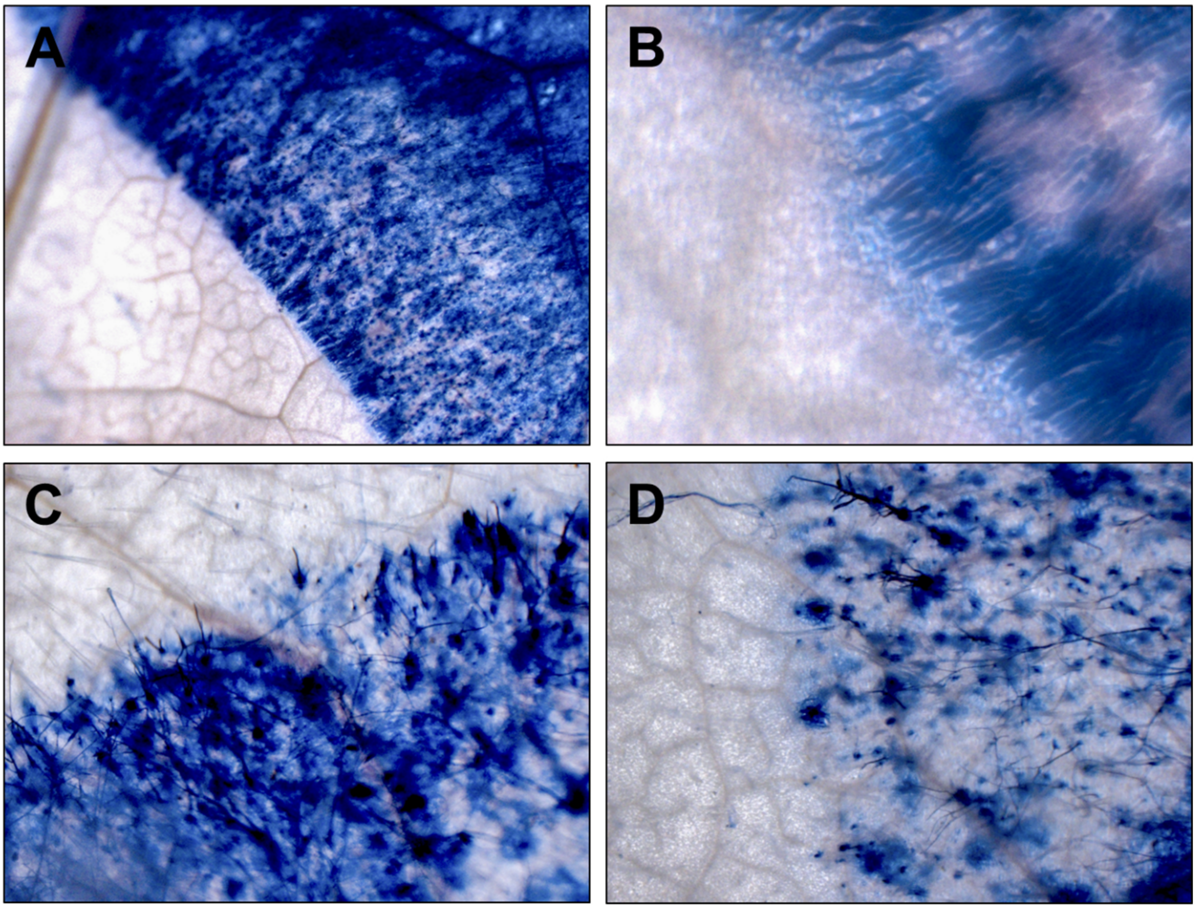

Figure S1 Trypan blue staining of inoculated leaves of **A – B** *B. oleracea* (BRA1909) and **C – D** *B. villosa* (BRA1896). A dense and compact structural fungal growth mainly within necrotic tissue was observed in leaves of the susceptible *B. oleracea*. In the resistant *B. villosa*, fungal expansion appeared less structured and focused mainly on the leaf surface with no delimited junction between healthy and infected tissue.

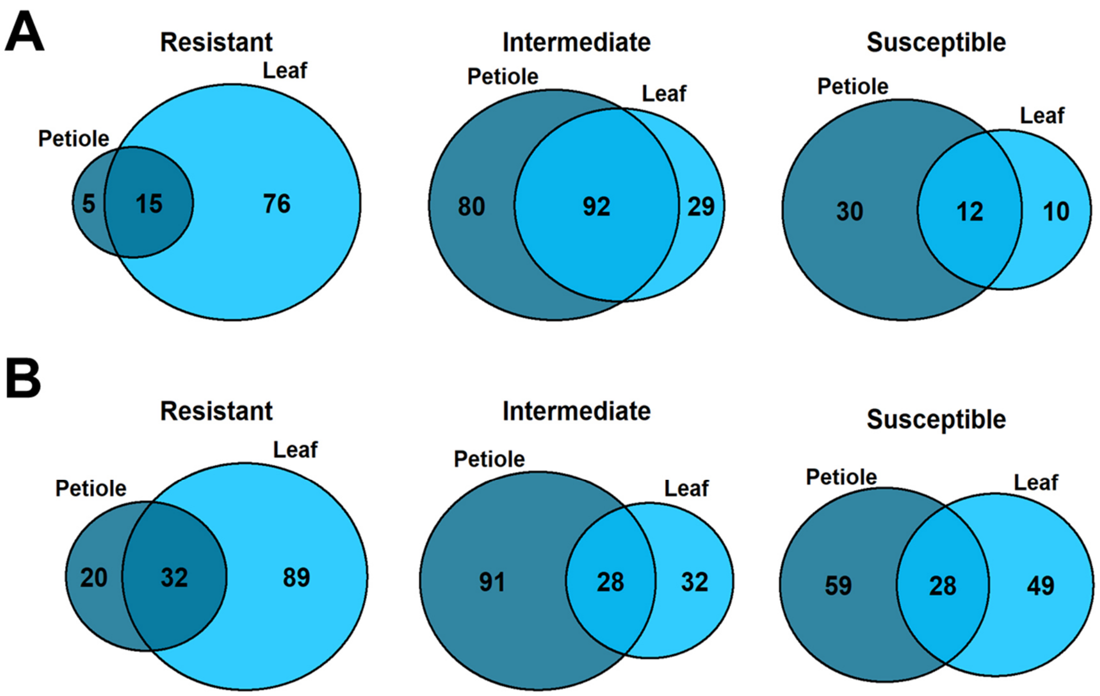

Figure S2 Venn diagram showing the proportion of F_2 individuals in **A** Population A and **B** Population B which were commonly classified as resistant, intermediate, and susceptible in the leaf- and petiole-assay. The grouping is based on the comparison to the resistant *B. villosa* (BRA1896) and the susceptible *B. oleracea* (BRA1909) parents in each population, respectively.

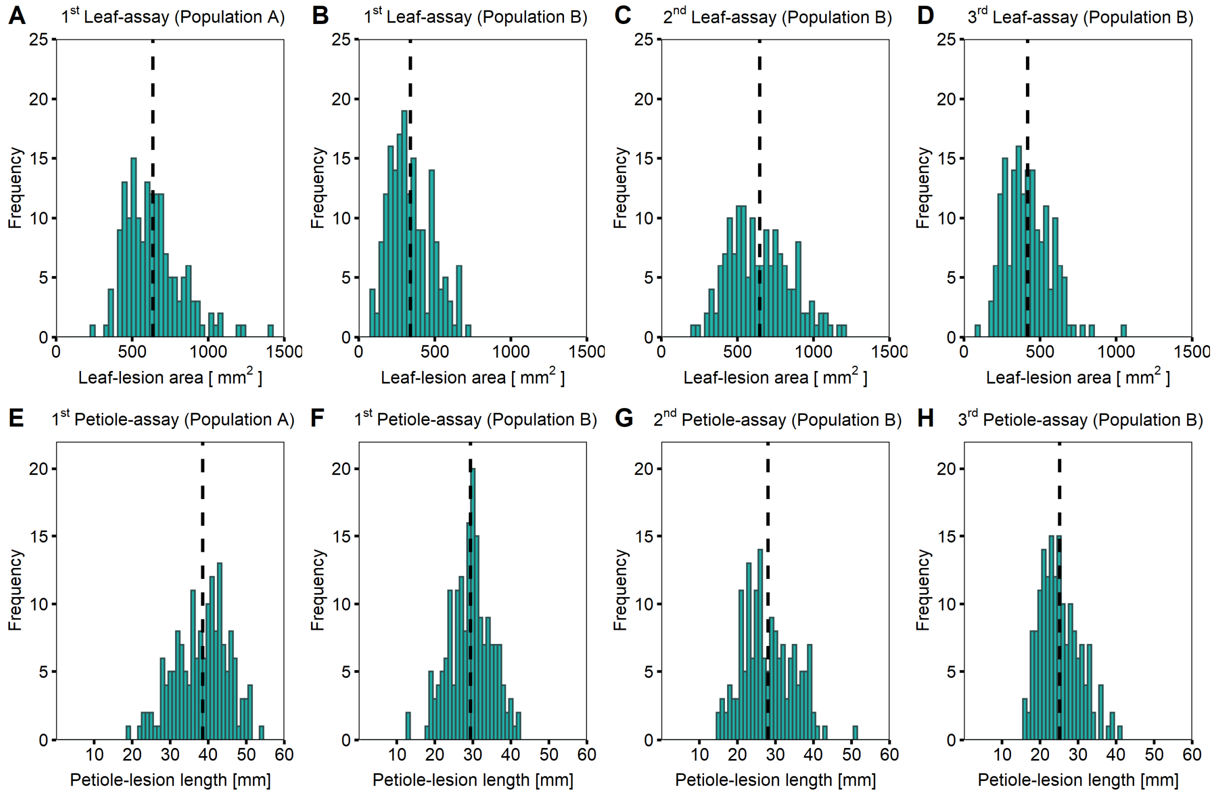

Figure S3 Lesion size distributions of genotyped F_2 plants. **A – D** Leaf-lesion size distributions of all genotyped F_2 individuals in **A** Population A and **B – D** in Population B. **E – H** Petiole-lesion size distributions of all genotyped F_2 individuals in **E** Population A and **F – H** in Population B.

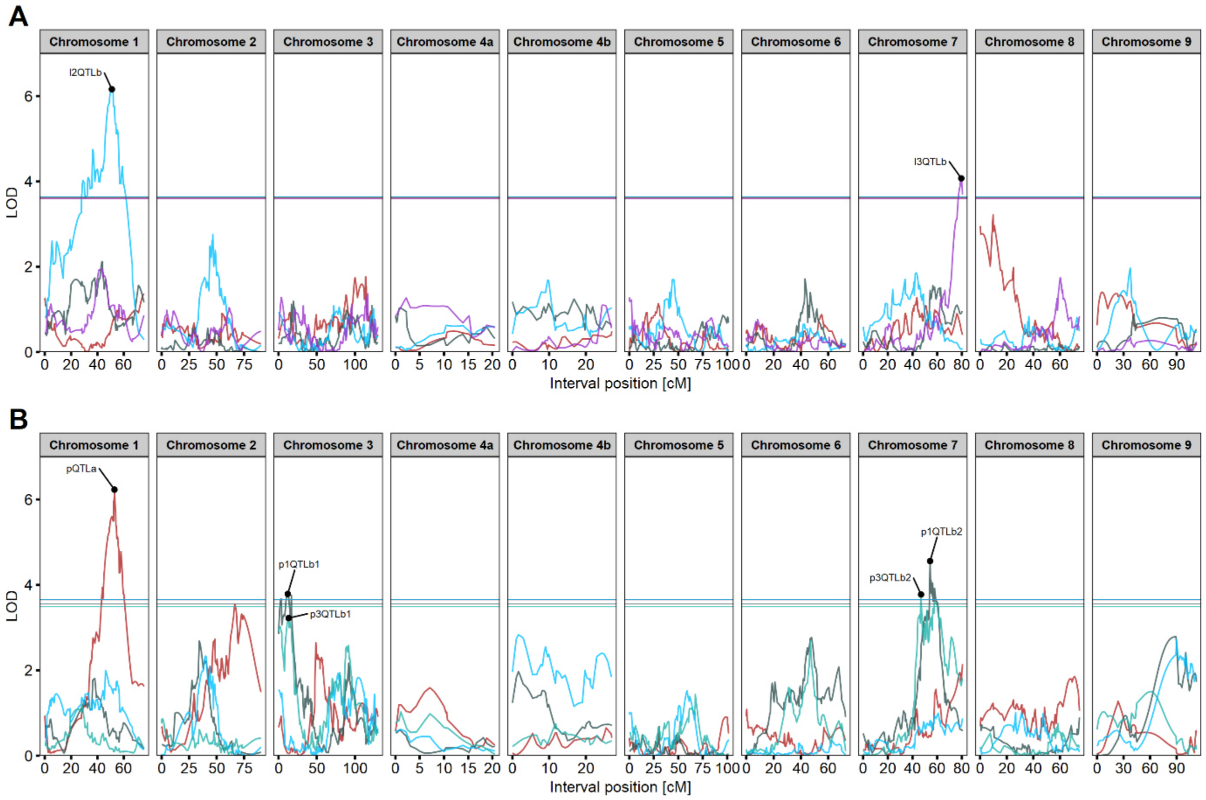

Figure S4 Logarithm of the odds (LOD) profiles in both mapping populations. Horizontal lines indicate the threshold for significance. Different assays are highlighted by different colors.

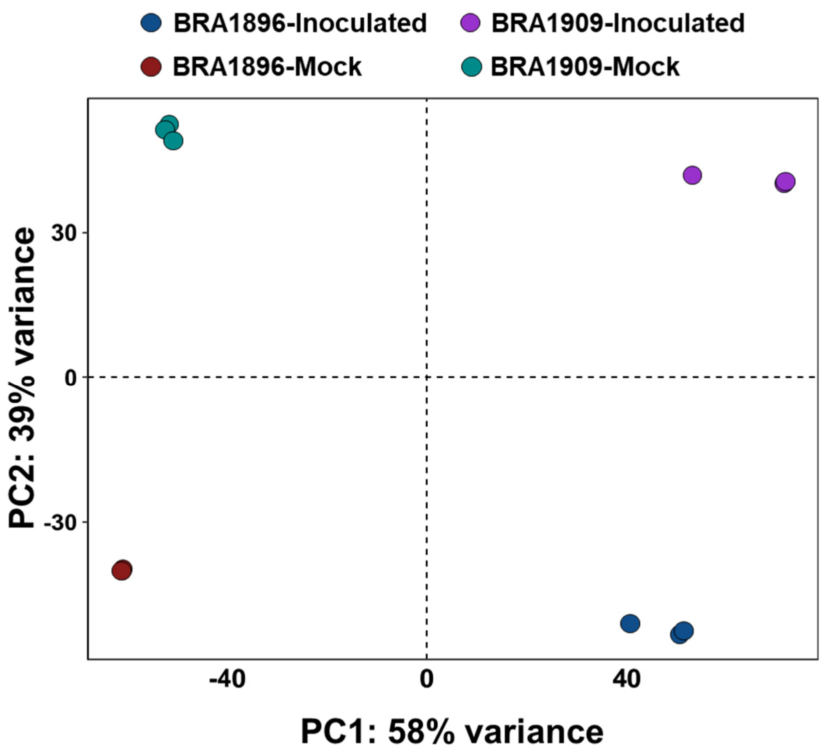

Figure S5 Principal component analysis (PCA) of RNAseq samples of *B. villosa* (BRA1896) and *B. oleracea* (BRA1909) after regularized log-transformation of the count matrix in DESeq2. Biological replications are highlighted by identical colors. PC1: variance between samples caused by species, PC2: variance between samples caused by the treatment.

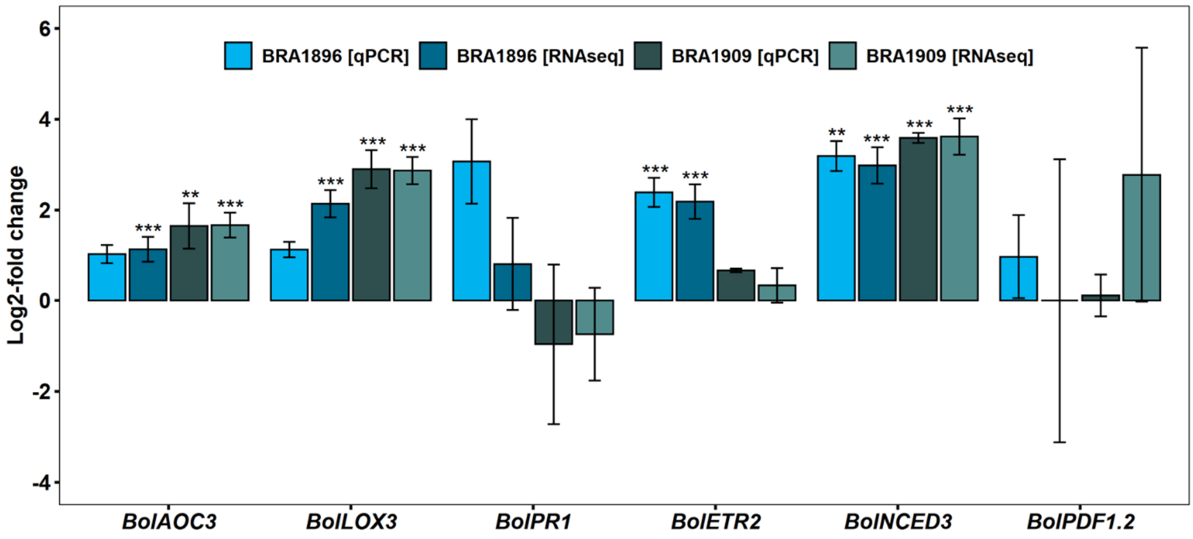

Figure S6 Marker gene expression comparison of reverse-transcribed quantitative PCR (RT-qPCR) and RNAseq data of resistant (BRA1896) and susceptible (BRA1909) petioles at 8 hours post inoculation (hpi) with Sclerotinia. Asterisks indicate a significant induction compared to the control sample. BRA1896 = *B. villosa*, BRA1909 = *B. oleracea*. RT-qPCR expression was log_2-transformed for a better comparison to the RNAseq. Genes in the RNAseq were as follows: *BolETR2* = Unigene.2465, *BolAOC3* = Bo9g075870, *BolLOX3* = Bo8g067210, *BolPDF1.2* = Bo2g086460, *BolNCED3* = Bo5g130280, *BolPR1* = Bo3g088360. Primers for RT-qPCR analysis are listed in Supplementary Table S1.

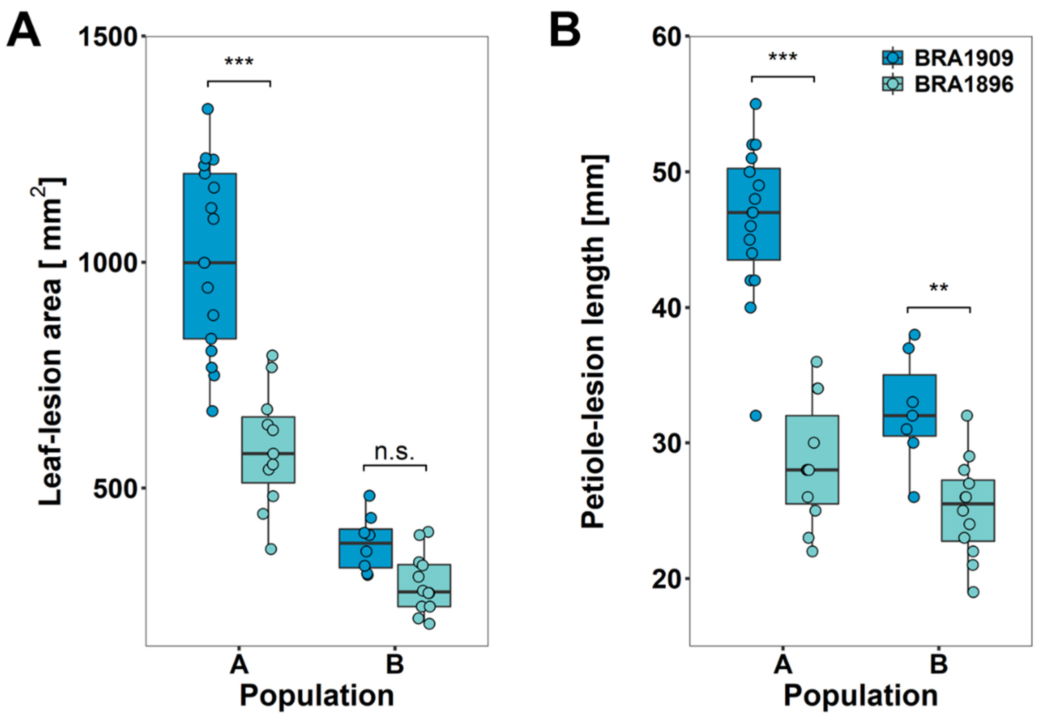

Figure S7 Lesion size comparison of the parental accessions in the two populations via the detached **A** leaf- and **B** petiole-assay at 2 days after Sclerotinia-inoculation under greenhouse conditions. Parental lesions in each population were compared with a linear model and multiple contrast tests. **P value < 0.01, ***P value < 0.001.
