# Supplementary material for: QTL mapping and transcriptome analysis identify novel QTLs and candidate genes in Brassica villosa for quantitative resistance against Sclerotinia sclerotiorum
Source: Theor Appl Genet. 2023 Mar 26;136(4):86. doi: 10.1007/s00122-023-04335-9 (PMC10040396; doi:10.1007/s00122-023-04335-9)
Supplement: Supplementary file 3 — Supplementary file3 (PDF 123 KB) [file 122_2023_4335_MOESM3_ESM.pdf]

Table S1 Primer used for real-time quantitative PCR gene expression analysis.

Gene	Forward [5' -> 3']	Reverse [5' -> 3']	bp	°C	Efficiency [%]
BolACT7	TCTACAACGAGCTCCGTGTTG	TGTGAGACACACCATCACCA	216	57	90
BolPR1	TCAGGTTGTTTGGAGAAAGTC	CATTAGTAAGGCTTCTCGTTCACA	126	57	103
BolETR2	CACTGCCATTGCACCTAGCTCTT	CAACTCCATTGATCCCAATCTGC	200	59	103
BolAOC3	GCTTTCTTCTGGAACTGGGGAAA	GATCGCCCGTGTAGAGTTTGTTG	181	60	99
BolLOX3	GTGAGGAACAAGAACAAGGAG	CGTTTTTGGATCAAGTTCGGTG	123	59	98
BolNCED3	ATGCTATTCTACGCCAGAGC	TGGGCGATCATTGTGGATTC	212	57	85
BolPDF1.2	TCTTGTTCTTTTCGCTGCTTTCG	TGCAAGATCCATGTCGTGCTT	155	59	88

Table S2 Summary of the genetic map.

Linkage group	Marker	Length [cM]	Ø-Distance	Max. gap [cM]
C01	121	75.4	0.63	7.8
C02	122	90.4	0.75	16.2
C03	233	129.9	0.56	7.9
C04a	29	20.5	0.73	3.2
C04b	36	27.1	0.77	2.6
C05	131	101.5	0.78	7.1
C06	131	72.7	0.56	5.1
C07	142	80.5	0.57	6.4
C08	139	74.6	0.54	6.1
C09	34	112.3	3.4	44.2
**Overall**	**1,118**	**784.9**	**0.71**	**44.2**

cM = centiMorgan

Table S3 Sequencing output and mapping rates for RNAseq samples of *B. villosa* (BRA1896) and *B. oleracea* (BRA1909).

Genotype	Treatment	Biological replicate	Clean reads [total]	*B. oleracea** Alignment rate [%]	Clean reads [not mapped to *B. oleracea*]	*S. sclerotiorum*** Alignment rate [%]
BRA1896	Mock	1	22,866,130	88.25	1,414,757	0
BRA1896	Mock	2	21,050,845	88.92	1,301,601	0
BRA1896	Mock	3	25,573,066	87.44	1,901,776	0.01
BRA1896	Inoculated	1	22,565,021	72.78	5,108,097	54.01
BRA1896	Inoculated	2	22,149,802	73.08	5,048,949	54.81
BRA1896	Inoculated	3	25,939,069	77.91	4,466,173	48.8
BRA1909	Mock	1	23,136,015	93.33	884,824	0.01
BRA1909	Mock	2	23,683,503	93.7	829,284	0
BRA1909	Mock	3	23,970,811	93.9	757,613	0.09
BRA1909	Inoculated	1	23,998,749	74.96	5,465,232	64.92
BRA1909	Inoculated	2	26,671,157	83.28	3,762,007	57.08
BRA1909	Inoculated	3	23,996,940	74.87	5,476,408	65.56
		**Overall**	**285,601,108**	**83.54**	**36,416,721**	**28.77**

* TO1000 reference genome (Parkin et al. 2014), ** Strain 1980 (Amselem et al. 2011).

Table S4 Specifically expressed genes involved in the immune response of *B. villosa*.

Gene_ID	Log_2-fold change		Homolog in *A. thaliana*	Putative gene
	BRA1896	BRA1909		
Unigene.14971	**1.18**	-0.91	AT3G55560	*AHL15*
Unigene.21095	**1.87**	0.29	AT1G44830	*ERF014*
Unigene.25806	**2.50**	0.16	AT5G47220	*ERF2*
Unigene.8766	**-1.24**	-0.89	AT2G37630	*MYB91*
Unigene.26057	**1.09**	0.66	AT3G25882	*NIMIN-2*
Unigene.38612	**-1.49**	0.24	AT1G09770	*CDC5*
Unigene.27241	**0.99**	-0.01	AT4G23570	*SGT1A*
Unigene.30096	**0.93**	0.36	AT3G55560	*AHL15*
Unigene.2328	**1.37**	0.28	AT1G61380	*SD1-29*
Unigene.33382	**1.47**	0.52	AT5G47220	*ERF2*
Bo4g178190	**1.15**	-0.36	AT2G32680	*RLP23*
Unigene.3661	**1.36**	0.50	AT5G03320	*PCRK2*
Unigene.22253	**1.09**	0.07	AT3G55560	*AHL15*
Unigene.7177	**0.61**	-0.32	AT5G66850	*MAPKKK5*
Unigene.19597	**0.59**	0.36	AT3G21650	*PP2A*
Unigene.29657	**1.08**	0.47	AT3G49120	*PRX34*
Unigene.32122	**1.40**	0.20	AT5G24090	*CHIA*
Unigene.13474	**0.91**	0.44	AT2G39660	*BIK1*
Trinity_DN2466_c0_g1_i7	**1.40**	0.00	AT3G05360	*RLP30*
Trinity_DN1154_c0_g1_i11	**3.06**	0.00	AT1G61380	*SD1-29*
Trinity_DN624_c0_g1_i2	**1.24**	0.00	AT5G03320	*PCRK2*
Trinity_DN787_c0_g3_i1	**4.25**	0.00	AT2G32680	*RLP23*
Trinity_DN1154_c0_g1_i51	**1.92**	0.00	AT1G61380	*SD1-29*
Trinity_DN787_c0_g3_i2	**2.24**	0.00	AT2G32680	*RLP23*
Trinity_DN1795_c0_g2_i9	**1.61**	0.00	AT3G09980	*ACIP1*

Significant log_2-fold changes are marked in bold (P adj. ≤ 0.05). Genes with a 'Trinity_' prefix were assembled without the reference genome.

Table S5 Ethylene-associated genes (iDEGs) with a significant difference in their expression between *B. villosa* (BRA1896) and *B. oleracea* (BRA1909).

**Gene_ID**	**Log$_2$-fold change**		**Homolog in *A. thaliana***	**Putative annotation**
	**BRA1896**	**BRA1909**		
Unigene.22432	**3.05**	0.81	AT3G59900	Auxin-regulated gene
Unigene.11755	**0.93**	-0.19	AT1G25560	Ethylene response DNA binding factor
Unigene.7022	**2.31**	0.35	AT5G61600	Ethylene response factor
Unigene.5240	**1.63**	0.15	AT1G70000	Myb-like domain transcription factor
Unigene.4348	-0.11	**3.16**	AT5G21960	Ethylene response factor
Unigene.24909	**6.10**	**2.82**	AT3G23220	Ethylene response factor
Unigene.21095	**1.87**	0.29	AT1G44830	Ethylene response factor
Unigene.17191	**1.92**	0.18	AT1G04310	Ethylene receptor
Unigene.25451	-0.12	**0.74**	AT5G67190	ERF/AP2 transcription factor
Unigene.2465	**2.19**	0.33	AT3G23150	Ethylene response factor
Unigene.11883	**3.46**	**1.77**	AT1G28370	Ethylene response factor
Unigene.5840	**1.12**	**2.60**	AT4G02380	Senescence-associated gene
Unigene.24908	**4.28**	**2.05**	AT3G23230	Ethylene response factor
Unigene.18643	**0.90**	**-0.63**	AT1G25560	Ethylene response DNA binding factor
Unigene.2457	**3.55**	0.40	AT3G23240	Ethylene response factor
Unigene.8085	-0.59	**-1.89**	AT5G59780	Myb domain protein
Unigene.24569	0.01	0.08	AT2G15900	Sorting nexin
Unigene.25806	**2.50**	0.16	AT5G47220	Ethylene response factor
Unigene.17161	**1.78**	-0.93	AT5G44210	Ethylene response factor
Unigene.21970	**1.08**	0.10	AT5G37260	Myb transcription factor
Unigene.12944	**1.29**	-0.03	AT2G40940	Ethylene response sensor
Unigene.33210	**3.68**	**1.80**	AT5G43410	Ethylene response factor
Trinity_DN1607_c1_g6_i1	**3.30**	-2.70	AT2G44840	Ethylene response factor

Significant log$_2$-fold change is marked in bold (P adj. ≤ 0.05).
